# Transcriptomic and differential gene analysis investigating the differences in biological behaviour between subtypes of feline alimentary lymphoma

**DOI:** 10.3389/fvets.2026.1764747

**Published:** 2026-05-29

**Authors:** Daniel McGilp, Paola Roccabianca, Lyndal Hulse, Rachel Allavena, Chiara Palmieri

**Affiliations:** 1 Veterinary Laboratory Services, School of Veterinary Science, The University of Queensland, Gatton, QLD, Australia; 2 Dipartimento di Medicina Veterinaria e Scienze Animali, Università degli Studi di Milano, Milan, Italy; 3 School of the Environment, The University of Queensland, Gatton, QLD, Australia

**Keywords:** bioinformatics, differential gene expression, lymphoma, oncogenomics, transcriptomics

## Abstract

Feline alimentary lymphoma encompasses a diverse group of neoplastic subtypes, each presumably arising from distinct cell populations and exhibiting significant variability in biological behaviour and prognosis. Whilst next-generation sequencing technologies have become a more common aid in the diagnosis, prognostication and treatment of lymphoma in humans, these techniques have been sparsely applied to investigate feline alimentary lymphoma. Specifically, current research in this field is mostly focused on differentiating indolent intestinal lymphomas from inflammatory enteropathies. The aim of the study was to apply transcriptomic analysis to identify differentially expressed genes that may correlate with the aggressiveness of various subtypes of feline alimentary lymphoma. Differential gene analysis was applied to RNA sequencing data from 19 cases of feline alimentary lymphoma split into five phenotypes (small T cell, B cell, CD56+ B cell, Large Granular T cell and Large Granular NK cell lymphomas) according to histomorphology and immunohistochemistry. The analysis identified overexpression of several genes involved in extracellular matrix remodelling, transcription factor expression and cellular division, signalling and metabolism in aggressive lymphoma types (ADAMTS14, ADAMTSl2, FOXI3, ELAVL2, CCR1, GCGR, NMUR1, MARC1, SLC29A4, CENPF, IGF2BP3); and overexpression of several tumour suppressor genes in the indolent lymphoma type (RAB17, SYNPO2, GRM4). The differential expression correlated with biologic behaviours. Future investigation into expression of these genes or gene products using molecular, immunohistochemical or *in-situ* hybridisation techniques could yield significant advancements in the prognostication of feline alimentary lymphoma as well as pave the way for the development of targeted immunotherapeutic strategies to aid in the treatment of specific feline alimentary lymphoma subtypes.

## 1 Introduction

Feline alimentary lymphoma (AL) is a common disease of cats arising from the transformation of native alimentary lymphocytes or their precursors and comprising a highly heterogenous group of neoplastic subtypes (1). Lymphocytes are derived from bone marrow haematopoietic precursor cells that differentiate into several subpopulations, including B, T and NK cells (2). Natural killer (NK) cells represent a distinct group of lymphoid cells that generally differentiate from lymphoid or more rarely myeloid precursors (3). These cells are defined by lack of T and B cell receptors, act in the innate immune responses against microbe-infected cells or neoplastic cells, and are sparsely distributed throughout body tissues, particularly mucosal surfaces (2–4).

In the alimentary tract, lymphoid cells populate unique anatomic niches depending on the subpopulation of lymphoid cells they are derived from (2, 4). Notably B and T lymphocytes form aggregates within the lamina propria and submucosa producing the gut-associated lymphoid tissues (GALT) that act as secondary lymphoid organs (2, 4), whilst NK cells are sparsely distributed within GALT (2, 4). Intraepithelial lymphocytes (IELs) comprise a separate population of T or NK alimentary lymphoid cells embedded individually within the gut mucosa between enterocytes (2, 4, 5). Previous studies have hypothesised that most alimentary B and T cell lymphomas arise from organised lymphoid tissues such as GALT, whereas aggressive large granular lymphomas arise from IELs (6–8).

Given lymphoma may emerge from any of these lymphocyte subpopulations or their precursors, the site of origin, cellular and architectural morphology, gene expression and biological behaviour varies amongst lymphoma types (9). Additionally, different types of lymphoma vary greatly in biologic aggressiveness and prognosis (2, 4, 9). Lymphoma classification is further complicated by the interaction of infectious agents, particularly viruses, as well as by genomic mutations and instability associated with neoplastic transformation (2, 4, 8, 9). As such, diagnosis of lymphoma can be difficult and classification systems continue to evolve as our understanding grows.

In the context of feline alimentary lymphoma, the human WHO/REAL lymphoma classification scheme has been widely adopted as a reference standard for lymphoma classification. Several studies have described the remarkable similarity of feline and human alimentary lymphoma types, allowing for the adaptation of this scheme to feline lymphoma (10–14). However, ongoing revisions of the WHO/REAL classification scheme and the increasing complexity of lymphoma classifications, has led to confusion in the application of this scheme to feline alimentary lymphoma, in particular regarding the so-called “low-grade alimentary lymphoma” (LGAL) (7, 15–19).

The increased availability of next-generation sequencing techniques in recent years has enabled the identification of unique gene expression panels across lymphoma subtypes, particularly in human oncology (9). In many cases, sequencing has elucidated aspects of lymphoma pathogenesis by identifying the upregulation and/or downregulation of genes that influence the behaviour of these neoplasms (9). Although specific gene mutations are not yet part of the diagnostic criteria for lymphoma in the human WHO/REAL classification scheme, the identification of altered gene expressions is influencing lymphoma prognostication and indeed, therapeutic approaches and as such it will likely be used in lymphoma diagnosis in the near future (9, 20–26). In contrast, the application of RNA sequencing and transcriptomics in the study of feline lymphomas is extremely limited with only two studies investigating the association between viral infections—such as Feline Leukaemia Virus (FeLV) and *Felis catus* gammaherpesvirus 1 (FcaGHV1)—and lymphoma being reported (27, 28). Moreover, there is limited application of transcriptomic or next-generation sequencing analyses to assist the differential diagnosis of inflammatory bowel disease (IBD) versus indolent lymphomas or to elucidate their underlying pathogenetic mechanisms. Research to detect genetic aberrations in feline lymphoma are generally limited to lymphoma cell lines or investigation of feline lymphoma tissues for specific genetic mutations with limited comparison amongst lymphoma types for prediction of prognosis (7, 27, 29).

The aim of this study was to employ transcriptomic and differential gene expression analysis to identify aberrant gene expression patterns between different types of feline alimentary lymphomas that may explain their differences in biological behaviour. To assess whether global transcriptomic profiles segregated according to lymphoma subtype, principal component analysis (PCA) was performed on normalised gene expression data. This approach allowed evaluation of sample clustering and assessment of overall variance between phenotypic groups (30). Differential gene expression analysis was subsequently conducted between predefined lymphoma subtypes to identify genes significantly associated with indolent versus aggressive phenotypes and with specific immunophenotypic categories. Gene ontology (GO) enrichment analysis was applied to differentially expressed genes to determine whether particular biological processes or molecular pathways were overrepresented in specific subtypes, thereby providing functional context to the observed transcriptional differences (31).

## 2 Materials and methods

### 2.1 Case selection and preparation

A total of 74 clinical cases of feline alimentary lymphomas from the archives of The University of Queensland and The University of Milan for which formalin-fixed paraffin embedded (FFPE) blocks of intestinal tissue were available were examined using histology and immunohistochemistry. This research received ethical approval from the UQ Animal Ethics Committee (Approval No. 2023/AE000379). The selected cases were classified as Small T cell Lymphoma, CD56− B cell lymphoma, CD56+ B cell lymphoma, NK cell Large Granular Lymphocyte (NK LGL) lymphoma, and T cell Large Granular Lymphocyte (T LGL) lymphoma by histopathology and immunohistochemistry. A subset of four cases from each classification (*n* = 20) were selected based on tumour density and tissue preservation within FFPE tissues by assessing the degree of autolysis, necrosis, artefact and haemorrhage.

Using a Leica RM2235 microtome (IL, United States), 10 tissue scrolls from each selected case were sectioned at 10 μm thick each and placed into labelled 1.5 mL non-lined DNAase/RNAase free Eppendorf microcentrifuge tubes. A new, disposable microtome blade was used for sectioning each case and between case sectioning the microtome was cleaned of debris and wiped down with 70% ethanol.

Labelled samples were transported to BGI genomics, Hong Kong for RNA sequencing using the DNBseq sequencing technology platform.

### 2.2 RNA sequencing

Total RNA was isolated from FFPE tissue sections using RecoverAll™ Total Nucleic Acid Isolation Kit for FFPE (Invitrogen) as per manufacturer’s instructions. Briefly, xylene was added to FFPE tissue sections to dissolve and remove paraffin residues and then removed by addition of ethanol, centrifugation and decantation. RNA was purified and concentrated using several cycles of centrifugation within RNeasy MinElute spin columns (Qiagen). Residual genomic DNA was eliminated by DNase treatment.

Following rRNA depletion using the Vazyme rRNA Depletion Kit (N408-C1, Vazyme-Nanjing, China), cDNA (complementary DNA) library preparation was performed by BGI Genomics (Shenzhen, China) using Hieff NGS C102P1 Ultima Dual-mode mRNA Library Prep Kit (Yeasen-Shanghai, China). Briefly, first-strand cDNA was synthesised from fragmented RNA under manufacturer-recommended reaction conditions, followed by second-strand synthesis and isothermal amplification to generate double-stranded cDNA. The resulting cDNA was fragmented, end-repaired, and adenylated at the 3′ ends prior to adaptor ligation. Adaptor-ligated products were amplified by polymerase chain reaction (PCR), denatured to yield single-stranded DNA, and enzymatically digested to remove uncyclised linear molecules. Circularised DNA molecules were subsequently subjected to rolling circle amplification to produce DNA nanoballs (DNBs) containing multiple copies of the original template. High-quality DNBs were loaded into patterned nanoarrays and sequenced on the DNBSEQ-G400 platform (BGI-Shenzhen, China) using combinatorial Probe-Anchor Synthesis (cPAS) chemistry with 50 base pair paired-end (50 PE) reads. Sequencing was successfully performed on 19 of 20 samples, with one sample from the NK LGL group excluded due to poor RNA quality.

Library preparation and sequencing quality control were performed by BGI Genomics using protocols optimised for fragmented RNA derived from FFPE tissues. Raw sequencing reads underwent filtering to remove adaptor sequences, low-quality reads, reads containing excessive ambiguous bases and short fragments prior to downstream analysis. Sequencing quality metrics demonstrated high base-calling accuracy across samples (Q20 > 97% and Q30 > 90%).

### 2.3 Bioinformatic analysis

**Figure 1:**
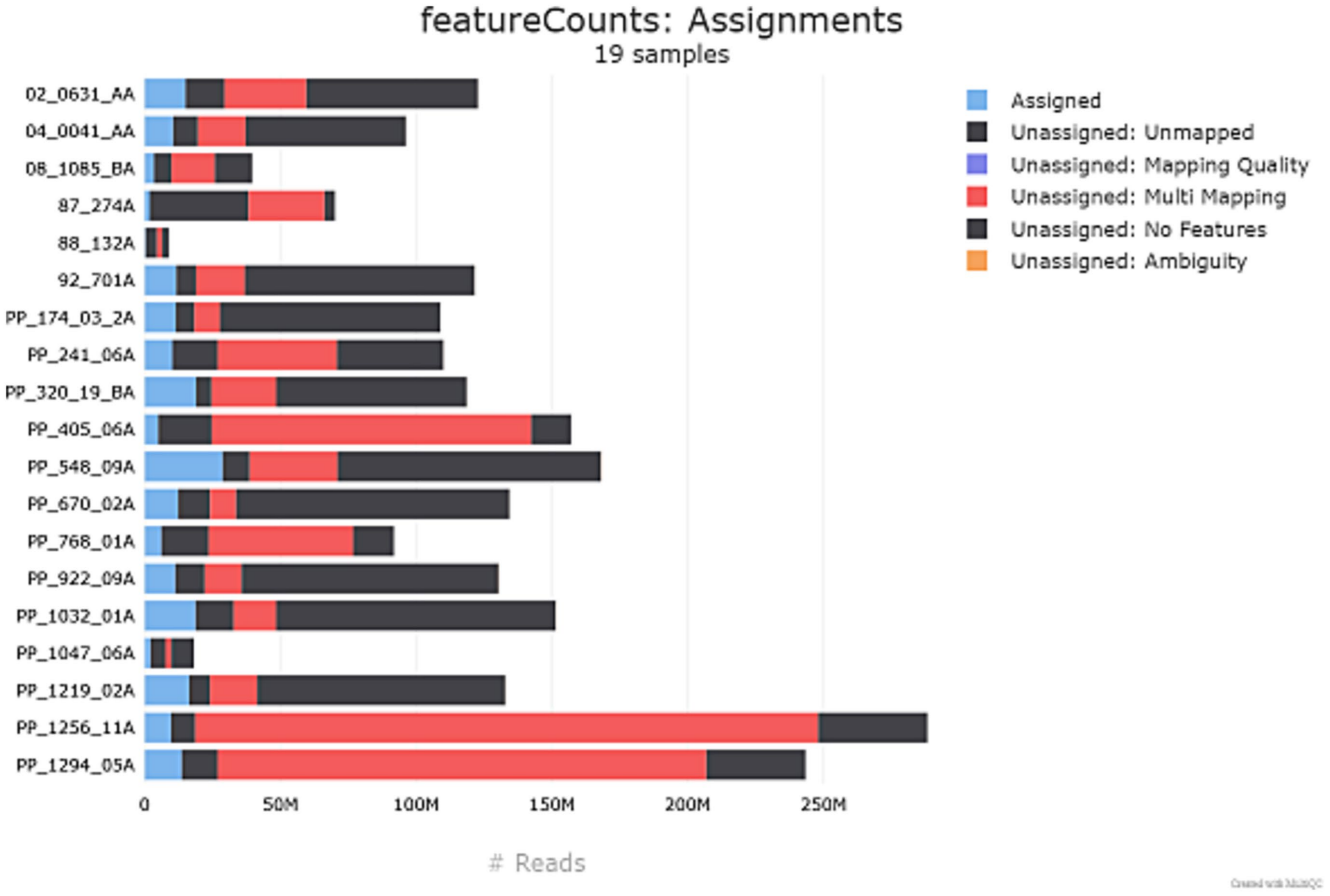
Bar chart displaying the assignment of reads for each case involved in the study based on the UCSC *Felis catus* 9 gene annotation file (https://hgdownload.soe.ucsc.edu/goldenPath/felCat9/bigZips/genes/).

Bioinformatic analysis was carried out using the Galaxy version 24.2 platform^1^ (32). Quality control was carried out using the Falco and MultiQC tools to visualise sequence quality (33, 34). Sequencing reads were trimmed and filtered using Cutadapt to remove adaptor sequences and low-quality bases (35). The resulting high-quality reads were then aligned to the *Felis catus* reference gene annotation (assembly felCat9) obtained from the University of California, Santa Cruz (UCSC) Genome Browser^2^ using RNA STAR (36). Further quality control was undertaken by assessing duplicate reads using MarkDuplicates; mapping genes to chromosomes with Samtools idxstats; evaluating gene body coverage using the Gene Body Coverage tool; and assessing read distribution across gene features with the Read Distribution tool (37, 38). Read counts per gene were quantified using featureCounts (39) (Figure 1). Differentially expressed genes (DEGs) were identified using DESeq2, with significance defined by an adjusted *p*-value < 0.02 and an absolute log_2_ fold change >1 (40). An adjusted *p*-value was used to define significance to limit the number of false positive cases that arise when analysing large data sets such as sequencing data (41). DEGs were determined both across all feline lymphoma types (small T-cell, CD56– B-cell, CD56+ B-cell, NK LGL, and T LGL) and through pairwise comparisons between groups, as well as by contrasting indolent small T-cell lymphoma with combined B-cell (CD56– and CD56+) and combined LGL (NK LGL and T LGL) groups. Heatmaps were generated for the top 10 DEGs in each comparison, and volcano plots were produced for all pairwise DEG analyses. Gene ontology enrichment analysis was performed on DEGs for all lymphoma types using the goseq tool and the *Felis catus* gene ontology file obtained from the National Centre for Biotechnology Information (NCBI)^3^ (42). Mapping and downstream analyses were also repeated using the NCBI *Felis catus* gene annotation file, which yielded highly comparable results to those obtained using the UCSC annotation (data not shown).

Principal component analysis (PCA) was performed using the Galaxy DESeq2 tool on the set of differentially expressed genes identified across all feline alimentary lymphoma cases involved in the study (40).

## 3 Results

### 3.1 Differentially expressed genes and gene ontology across all feline alimentary lymphoma groups

**Figure 2:**
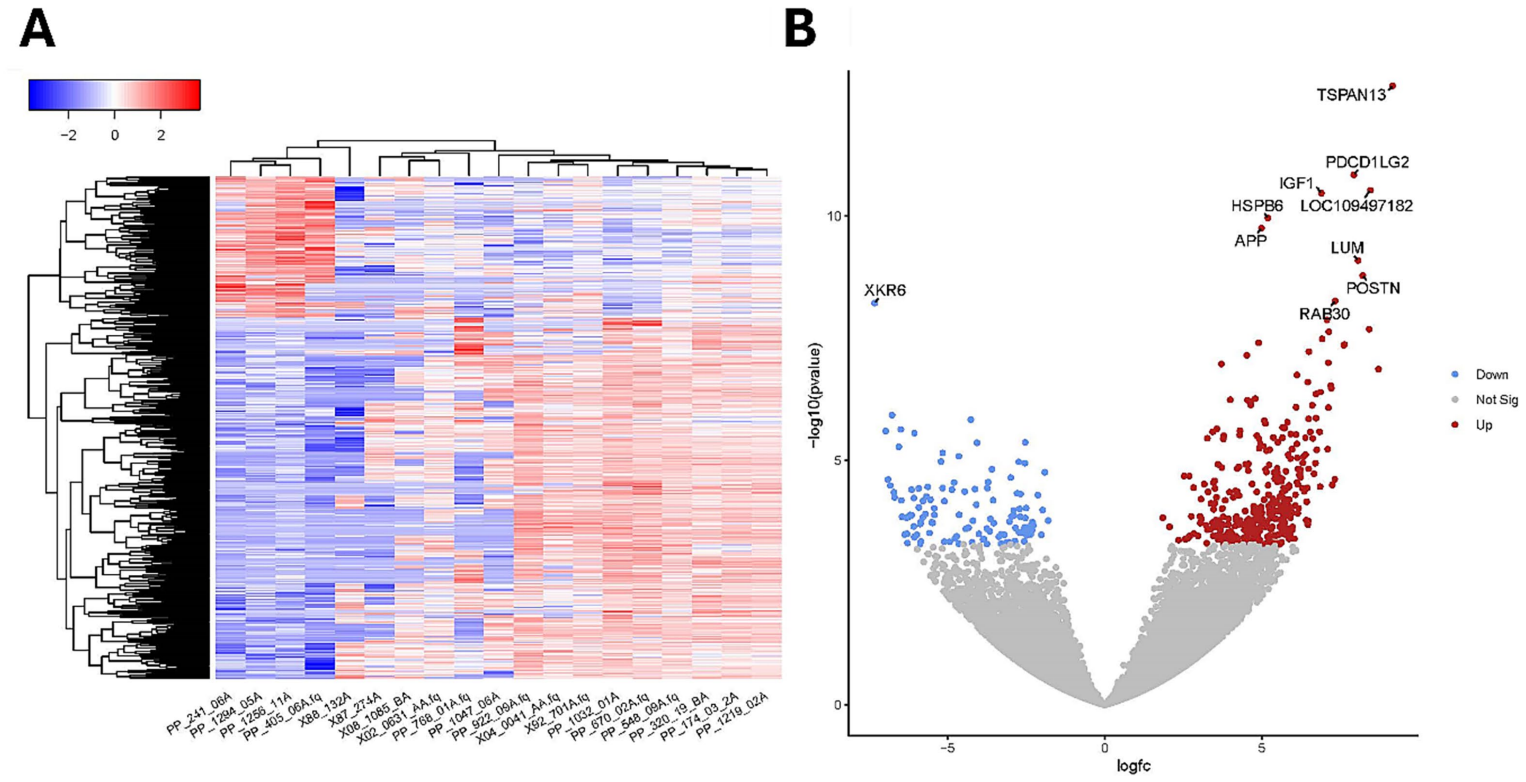
Heat map **(A)** and volcano plot **(B)** displaying all differentially expressed genes across all feline alimentary lymphoma groups, where red indicates overexpression and blue indicates underexpression of each gene. Colour in the volcano plot **(B)** indicates that the gene corresponding to that point is significantly over or under expressed.

Across all feline alimentary lymphoma groups, 787 DEGs were identified,(adjusted *p*-value <0.05, fold change ≥2), including 567 upregulated and 220 downregulated (Figure 2).

**Figure 3:**
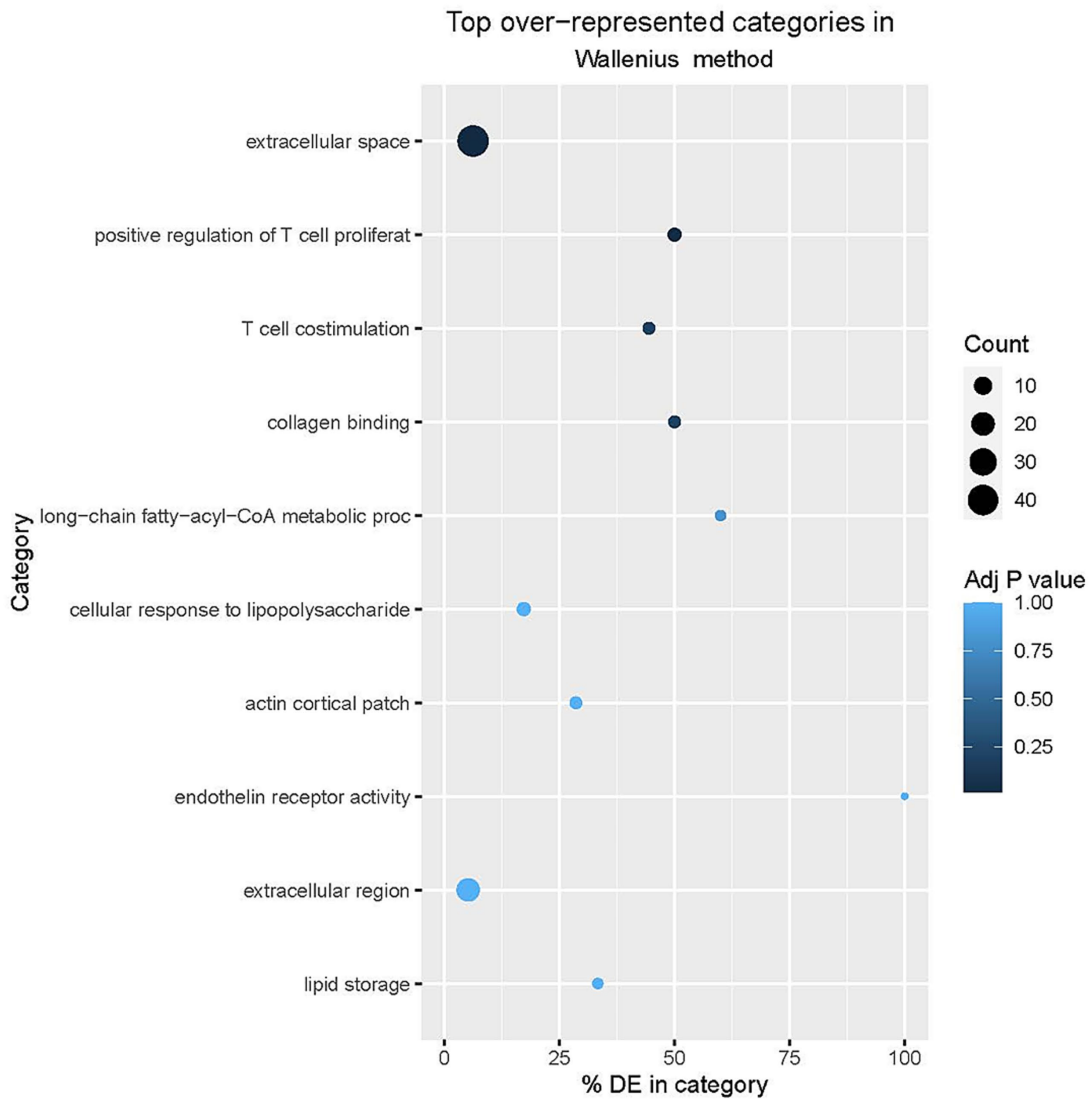
Gene ontology chart demonstrating the categories of gene functions over-represented by differential gene analysis amongst all types of feline alimentary lymphoma included in this study.

Gene ontology enrichment analysis indicated overrepresentation of pathways related to extracellular matrix organisation, T cell activation/proliferation, collagen binding, lipid metabolism, actin cortical patch development, endothelin receptor activity and cellular responses to lipopolysaccharide (Figure 3).

### 3.2 Principal component analysis and differential gene expression of outlier cases

**Figure 4:**
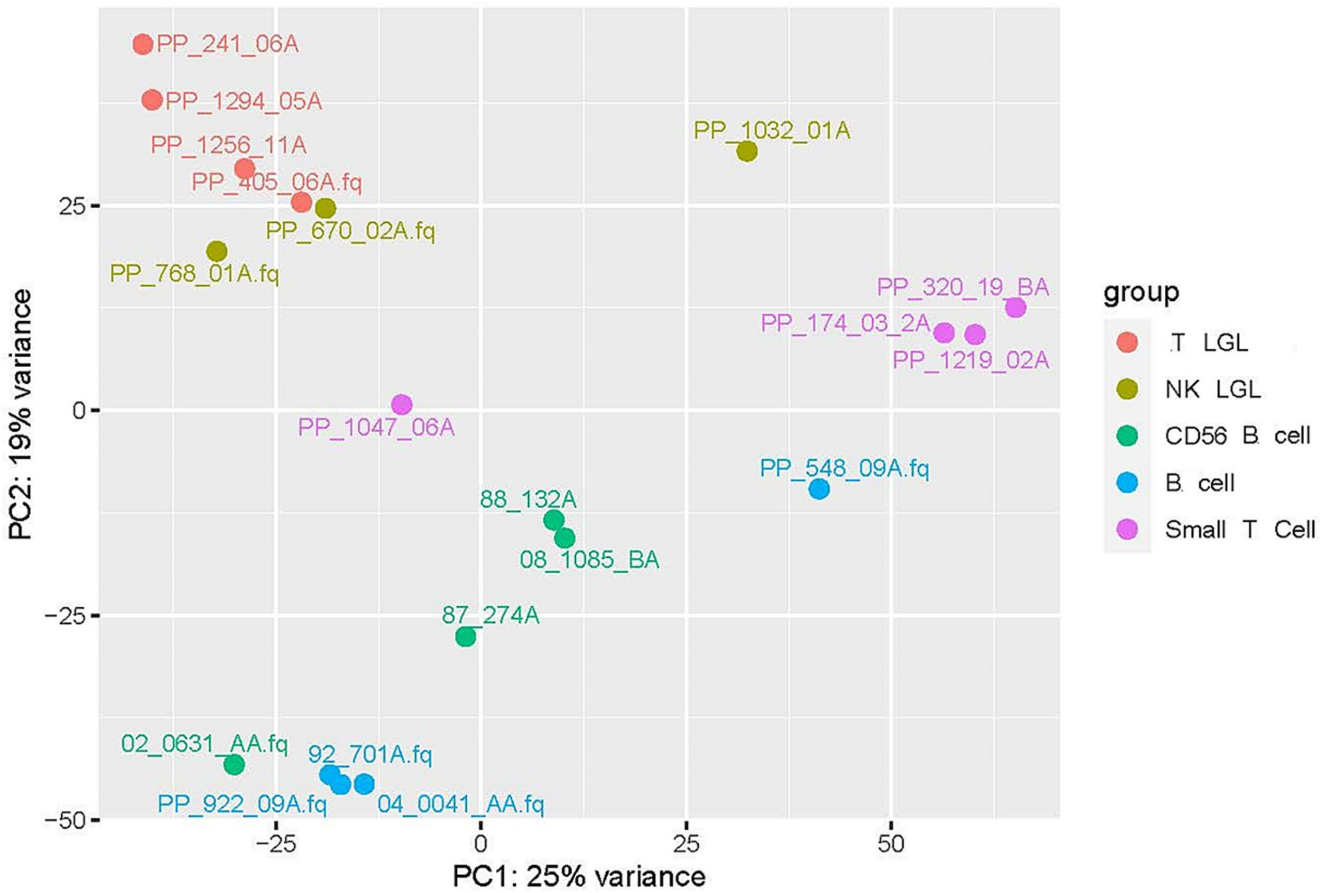
PCA biplot displaying variance between each case of feline alimentary lymphoma with clustering of cases by the lymphoma subtype as indicated by case colour.

Principal component analysis (PCA) demonstrated a clear separation amongst the five feline lymphoma subtypes (Figure 4). The CD56+ and CD56− B cell lymphoma groups overlapped, as did the T cell LGL and NK cell LGL lymphoma groups, whereas. The small T cell lymphoma group formed a distinct cluster. Four outliers were identified; one in each of the small T cell lymphoma (PP_1047_06A), NK LGL lymphoma (PP_1032_01A), CD56+ B cell lymphoma (02_0631_AA) and CD56− B cell lymphoma (PP_548_09A) groups. The small T cell lymphoma outlier showed markedly lower gene counts compared to other cases within the same group (Figure 1). No significant differential expression (*p* > 0.05) was detected for the NK LGL a and CD56+ B cell outliers.

In contrast, the CD56− B cell outlier exhibited 97 DEGs compared with other cases of the same subtype (adjusted *p*-value <0.02, a fold change ≥2), including 58 upregulated and 39 downregulated genes. Upregulated genes amongst the top DEGs included MUC17, MUC2, KRT8, UNC79, HNF4A, CLDN2, BPI, and CLCA4, whilst RGS13, BEND4, FCRL1 and GABRA4 were downregulated (Supplementary Table 1).

### 3.3 Differentially expressed genes across feline alimentary lymphoma group pairs

Pairwise comparisons between the five feline alimentary lymphoma subtypes revealed substantial transcriptional differences (Supplementary Figures 5–14). The number of differentially expressed genes (DEGs) varied widely between comparisons, ranging from 56 genes between CD56+ B cell and NK LGL lymphomas to 3,500 genes between CD56− B cell and T LGL lymphomas (Table 1).

**Table 1:** Summary of pairwise DEG comparisons between feline alimentary lymphoma subtypes.

Comparison	Total DEGs	Upregulated	Downregulated	Representative upregulated genes
Small T vs. CD56− B	680	351	329	ADGRG5, CCR9, LAT, ITGB7, ITK
Small T vs. CD56+ B	345	321	24	CPNE5, TCF7, CCR9, RNF125
Small T vs. NK LGL	226	161	65	CD4, FCRL1, RNF39, MS4A1
Small T vs. T LGL	2,379	1,611	768	JCHAIN, CD4, PDCD1LG2
CD56− B vs. CD56+ B	112	106	6	RNF125, FAM210A, RPH3A
CD56− B vs. NK LGL	357	189	168	MS4A1, CD19, PAX5
CD56− B vs. T LGL	3,500	2,372	1,128	MS4A1, FCRL1, POU2AF1
CD56+ B vs. NK LGL	56	14	42	MS4A1, PAX5
CD56+ B vs. T LGL	374	145	229	POU2AF1, FCMR, FCRL3
NK LGL vs. T LGL	256	193	63	CSN2, APP, PDCD1LG2

Comparisons involving small T cell lymphoma consistently demonstrated marked transcriptional divergence from other subtypes. Relative to CD56− B cell lymphoma, 680 DEGs were identified, whilst 345 DEGs and 226 DEGs were identified when compared with CD56+ B cell and NK LGL lymphomas, respectively. The largest difference involving small T cell lymphoma occurred in comparison with T LGL lymphoma, where 2,379 DEGs were detected. Genes associated with T-cell signalling and activation, including ADGRG5, CCR9, LAT, ITGB7, ITK, TCF7 and PRKCQ, were amongst the most upregulated in the small T cell group (Supplementary Tables 2–5). In contrast, the TRG gene was significantly overexpressed in the T LGL group and not expressed in small T cell lymphoma.

Comparisons between B cell lymphoma subtypes showed relatively limited transcriptional differences, with 112 DEGs identified between CD56− and CD56+ B cell lymphomas. In contrast, comparisons between B cell and LGL lymphomas demonstrated larger transcriptional differences. B cell lineage genes, including MS4A1, CD19, PAX5, FCRL1, POU2AF1 and TNFRSF17, were consistently upregulated in B cell lymphomas relative to LGL lymphomas.

The largest transcriptional divergence overall occurred between CD56− B cell and T LGL lymphomas, with 3,500 DEGs, whereas relatively few differences were observed between CD56+ B cell and NK LGL lymphomas (56 DEGs). Differences between NK LGL and T LGL lymphomas included 256 DEGs, with genes such as CSN2, APP, PDCD1LG2, IGF1, GCM2 and RAB30 upregulated in the NK LGL group.

Detailed DEG counts and representative genes for each comparison are summarised in Table 1, with full gene lists provided in Supplementary Tables 2–11.

### 3.4 Differentially expressed genes between *indolent* small T cell lymphoma, *aggressive* B cell lymphomas (CD56− B cell and CD56+ B cell groups) and *highly aggressive* large granular lymphomas (NK LGL and T LGL groups)

Comparison of indolent small T cell lymphomas and highly aggressive Large Granular Lymphocyte lymphomas (comprising NK LGL and T cell LGL) identified 279 DEGs (adjusted *p*-value <0.05, fold change ≥2) with136 DEGs upregulated and 143 downregulated genes (Supplementary Figure 15). Amongst the most upregulated genes in the small T cell lymphoma group were.

FCRL1, MS4A1, CD4, KCNK10, CD40LG, MYH11, PLB1, TNFRSF17, RAB17 and SYNPO2 whereas CC1H1orf167, ADAMTS14, FOXI3, CCR1 and GCGR were downregulated (Supplementary Table 12).

Comparison of indolent small T cell lymphomas group and aggressive B cell lymphomas (comprising CD56+ B cell and CD56− B cell lymphoma groups)identified 182 DEGs, with 120 DEGs upregulated and 62 DEGs downregulated genes (Supplementary Figure 16).

Upregulated genes in the small T cell group included LAT, CCR9, AIFM3, GRM4, ADGRG5, PNMA6A, GDF6 and TARP, whilst 4 known genes (IGF2BP3, CENPF, MEF2B and ANKRD34C) were downregulated, 7 unassigned loci were upregulated and 1 unassigned locus was downregulated (Supplementary Table 13).

Finally, comparison of aggressive B cell lymphomas and highly aggressive LGL lymphomas identified 606 DEGs, including 184 upregulated and 422 downregulated genes. B cell lineage genes including FCRL1, MS4A1, PAX5, CD19, MEF2B, EAF2, FCRLA and TNFRSF17 were upregulated in B cell lymphomas, whereas genes including AIFM3, GCGR, TARP, MARC1, TSGA10IP, SLC29A4, GREB1, NMUR1, ELAVL2 and ADAMTSL2 were downregulated (Supplementary Table 14).

## 4 Discussion

### 4.1 Principal component analysis (PCA)

PCA further supported the differentiation of feline intestinal lymphomas into the five subtypes recognised morphologically and immunophenotypically.

Interestingly, PCA identified 1 outlier in each of the small T cell lymphoma, CD56− B cell lymphoma, CD56+ B cell lymphoma and NK LGL lymphoma subtypes. Read counts were significantly reduced (by more than two orders of magnitude) in the aberrant small T cell lymphoma outlier (Figure 1), suggesting RNA degradation in the sample as the most likely cause of aberrance in the PCA biplot. Outliers in the NK LGL and CD56+ B cell lymphoma group revealed no statistically significant difference in gene expression and thus were considered artefactual.

The outlier case in the CD56− B cell lymphoma group differentially expressed 97 genes compared to the rest of the CD56− B cell cluster. Notably, several genes in the outlier were either not expressed, or minimally expressed in the other three clustered cases. Several of these overexpressed genes are classically associated with intestinal epithelial cells, particularly colonic epithelium, including KRT8, HNF4A, CLDN2, MUC2, MUC17, BPI and CLCA4. Most of these genes are overexpressed by human enterocytes during inflammatory conditions or other insults causing epithelial injury (such as adjacent neoplastic infiltration) (43–49). This could suggest that the outlier case reflects the origin from a more inflammatory microenvironment. Alternatively, overexpression of these genes could suggest that lymphoma in the aberrant case originated in the colon given the strong expression of these genes by colonic epithelium. Unfortunately, anatomical location data were not available for these cases and so this second hypothesis could not be confirmed. On further analysis, three genes were underexpressed by the outlier CD56-B cell case, namely RGS13, BEND4 and FCRL1. Regulator of G protein signalling 13 (RGS13) and Fc receptor like 1 (FCRL1) both encode surface signalling molecules involved in the development and differentiation of B cells. Underexpression of RGS13 in this outlier may indicate that this case is not a diffuse large B cell lymphoma (DLBCL), as the other clustered cases (50). Expression of FCRL1 is negatively correlated with prognosis in B cell neoplasms (51). Similarly overexpression of the BEN containing domain 4 (BEND4) gene in DLBCL is associated with poorer prognosis (26). Hence, underexpression of FCRL1 and BEND4 by the outlier case may suggest a less aggressive form of DLBCL compared to the other clustered cases. This could partially explain the closer association of the CD56− B cell lymphoma outlier case with the less aggressive small T cell lymphoma group on the PCA biplot.

### 4.2 Gene ontology (GO)

In our study, using the goseq tool in Galaxy, we identified gene ontology terms over-represented across all feline alimentary lymphoma cases (Figure 3) (42). Across lymphoma subtypes, DEGs were mainly involved in the extracellular space/region, collagen binding, T cell stimulation/proliferation, actin cortical patch, metabolism of fatty acids/lipid storage and endothelin receptor activity. This may suggest that differences in tumour aggressiveness relies in part on tumour interactions with the extracellular matrix (ECM) as well as metabolic alterations, as reported in many aggressive human and murine neoplasia (colonic, mammary, pancreatic, pulmonary, prostate, ovarian carcinomas and melanoma) (21, 52–55). On the other end, the overrepresentation of T cell stimulation/proliferation pathways across different feline alimentary lymphoma types may be more reflective of differences in immunophenotype rather than indicators of true biologic aggressiveness.

### 4.3 Indolent small T cell lymphoma compared to highly aggressive large granular lymphocyte (LGL) lymphoma

Compared to small T cell lymphoma, LGL lymphomas overexpressed disintegrin-like and metallopeptidase with thrombospondin type 1 motif (ADAMTS14), Forkhead box protein I3 (FOXI3) and Chemokine receptor 1(CCR1). All these genes.

modulate cell adhesion, cell fusion, cell migration, extracellular matrix structure and proteolysis (ADAMTS) (56–59), as well as lymphovascular invasion and modulation of the tumour microenvironment (FOXI3) (21) and production of ligands that drive neoplastic growth (CCR1) (60, 61), which may contribute to their aggressive phenotype and short survival times of affected patients. In addition, Tumour associated macrophages (TAMs) are also frequently implicated in tumour growth and progression and generally have high expression of CCR1 (62). Indeed, CCR1 inhibition in some human lymphomas appears to reprogram TAMs, leading to more favourable prognoses (62).

The glucagon receptor gene (GCGR) was also overexpressed within the LGL group compared to small T cell lymphoma. Expression of GCGR appears to have a complex relationship in different types of cancer, however, it has not been exclusively studied in lymphomas (55, 63, 64). It may be hypothesised that overexpression of the glucagon receptor by LGLs enables survival and growth of neoplastic cells in a low nutrient environment, possibly aiding their rapid growth or distant metastasis.

Conversely, RAS Oncogene Family 17 (RAB17) and synaptopodin 2 (SYNPO2) were underexpressed in LGLs, potentially further supporting increased aggressiveness. RAB17 appears to act as a tumour suppressor gene across many types of human cancer, although its expression in lymphoma has not been described (65), and its underexpression of RAB17 has been associated with increased neoplastic aggression, invasion, angiogenesis and accelerated tumourigenesis through the STAT3/HIF-1a/VEGF axis (65, 66). SYNPO2 is another tumour suppressor gene across many cancer types through the PI3K/AKT/mTOR and Hippo signalling pathway regulation (67).

Small T cell lymphoma group overexpressed CD4 and the cluster of differentiation 40 ligand (CD40L)—gene encoding for a protein expressed by activated T cells, generally with a CD4+ immunophenotype (68)—suggesting a CD4+ T cell origin for these tumours. Interestingly the current WHO/REAL description for indolent T cell lymphoma of the gastrointestinal tract describes most cases as being CD4+ in contrast to monomorphic epitheliotropic intestinal T cell lymphoma (MEITL) and enteropathy associated intestinal T cell lymphoma (EATL) that are generally CD4-negative (9). This supports the idea that feline small T cell lymphomas are analogous to the human indolent T cell lymphoma of the gastrointestinal tract (iTL-GI). However, the intestinal T-cell lymphoma not otherwise specified (ITCL-NOS) category (that may be either CD4+ or CD4-/CD8-) should also be considered in the investigation of CD4+ cases (9).

Other differentially expressed genes with unclear functional significance—Potassium channel subfamily K member 10 (KCNK10), Myosin Heavy Chain 11 (MYH11) and Phospholipase B1 (PLB1)—were overexpressed in small T cell lymphomas, although their role in tumour biology remains uncertain (69–72).

The Small T cell lymphoma group in this study overexpressed several B cell-associated genes including MS4A1, FCRL1 and TNFRS17. Histological and immunohistochemical examination of these cases, identified CD20+/PAX5+ B cells within lymphofollicular structures adjacent to the neoplastic T cell infiltrates and were interpreted as native reactive B cells responding to the neoplastic environment. Hence the overexpression of genes correlating to B cell markers in the small T cell lymphoma group compared to LGLs is most likely due to expression of genes in this reactive, non-neoplastic population of B cells. Furthermore, the overexpression of CD40L in the small T cell lymphoma population could at least partially explain the aggregation of reactive B cells adjacent to these neoplasms. In future studies, laser capture microdissection could be employed on FFPE tissues to isolate purely neoplastic cell populations for RNA sequencing modalities that would reduce the impact of native or reactive lymphocytes “contamination” of RNA sequencing samples (73).

### 4.4 Indolent small T cell lymphoma vs. aggressive B cell lymphoma

Compared to small T cell lymphomas, aggressive B cell lymphomas overexpressed Centromere protein F (CENPF) and Insulin-like growth factor 2 mRNA binding protein 3 gene (IGF2BP3), potentially underpinning higher tumour aggressiveness.

CENPF overexpression is a feature of many biologically aggressive human cancers including lymphoma, particularly diffuse large B cell lymphoma inducing chromosomal instability (25, 74–78). Interestingly, Miki et al. (1) demonstrated centrosome amplification and chromosomal instability in five feline lymphoma cell lines *in vitro*, suggesting that dysregulation of centriolar protein related genes such as CENPF may play a mechanistic role in tumour progression for this IGF2BP3 is an oncofoetal protein that is not normally expressed or expressed at low levels in adult tissues; however expression is frequently reactivated during neoplastic transformation and cancer progression (79). Several human lymphoid neoplasms overexpress IGF2BP3 including B cell leukaemia and mantle cell lymphoma (20, 79, 80). *In vitro* inhibition of this protein has proven effective in killing some murine B cell leukaemia cell lines (20, 80), making it a compelling avenue for translational research into feline large B cell lymphomas.

Other genes with uncertain biological relevance were Glutamate metabotropic receptor 4 (GRM4) and Myocyte enhancer binding factor 2B (MEF2B), respectively downregulated and upregulated in B cell lymphomas. GRM4 is a tumour suppressor gene (81, 82) with low expression associated with poorer prognosis in human gliomas and mammary carcinomas (81, 82). MEF2B, a transcriptional activator of the BCL6 proto-oncogene in normal germinal centre B-cells, is p is overexpressed in B cell lymphomas, reflecting cellular origin. In human lymphoma, MEF2B dysregulation can promote neoplastic progression (22, 24), suggesting it may contribute to the aggressive phenotype of feline B-cell lymphomas. When interpreting DEGs between small T-cell and B-cell lymphomas, the presumed cell of origin should be taken into consideration. Predictably, several genes overexpressed in the small T cell lymphoma group, including TARP (T cell receptor gamma chain alternate reading frame protein), LAT (linker for activation of T cells) and AIFM3 (apoptosis inducing factor, mitochondria associated 3), are canonically expressed by T cells but not known to be natively expressed by normal B cells. These genes included. TARP, normally expressed by CD4+ T cells, CD8+ T cells, and NK cells, is under investigation as an immunotherapy target in human cancers, suggesting potential translational relevance in feline T-cell lymphomas (83).

Finally, the significance of GDF6 and CCR9 overexpression in the small T cell lymphoma group compared to B cell lymphomas is unclear. In human cancers, GDF6 is over and underexpressed according to the cancer types (84) and may influence immunotherapy response, since high GDF6 expression is associated with resistance to anti-PD1(programmed cell death protein 1) immunotherapy and simultaneous sensitivity to anti-PD-L1 (programmed cell death ligand 1) immunotherapy (84). Further investigation into the expression of GDF6 in feline lymphomas is required to elucidate its role in tumour aggression and immunotherapeutic potential.

### 4.5 Aggressive B cell lymphoma vs. highly aggressive large granular lymphocyte (LGL) lymphoma

Several genes related to the extracellular matrix regulation and cellular metabolism were overexpressed in LGL lymphomas compared to the B cell lymphoma group, including ADAMTSL2, NMUR1, ELAVL2, GCGR, SCL29A4 and MARC1. ADAMTS genes (including ADAMTS-like 2), involved in ECM remodelling and angiogenesis (56–58, 85), is associated with shorter survival times in human cancers (including several lymphoma types) and interestingly appeared to be particularly overexpressed in tumours of the digestive system (85, 86), potentially explaining part of the increased aggressiveness of LGLs.

Neuromedin U receptor 1 (NMUR1) and ELAV-like RNA-binding protein 2 (ELAVL2) overexpression in many human cancer types has been linked to tumourigenesis with different mechanisms including invasion, migration and metastasis (NMUR1) (87–89) and resistance to apoptosis (ELAVL2) (90, 91). In human lymphoma, leukaemia and oral carcinomas, cytoplasmic ELAVL2 protein expression has been demonstrated to be a poor prognostic indicator (90–93).

As discussed above, it is possible that GCGR expression in the LGL group confers more rapid growth of neoplastic cells in an otherwise inhospitable environment (54). The role of I SLC29A4 overexpression in the LGL group is unclear, but may enhance nucleic acid synthesis, potentially driving neoplastic progression. There is limited investigation into the significance of MARC1 overexpression in neoplasia, however a study by Klopp et al. identified MARC1 overexpression linked to chemoresistance in human leukaemia a (94).

Two additional genes—testis specific 10 interacting protein (TSGA10IP) and Growth regulation by oestrogen in breast cancer 1 (GREB1)—were also overexpressed by the LGL group, although their biological significance is unclear. The TSGA10IP gene is normally expressed by T, B and NK cells and is involved in cilium organisation (23, 71, 95), whilst GREB1, a coactivator of the oestrogen receptor, has been reported in several carcinoma, but not in lymphoid neoplasms (96).

Many other DEGS likely reflect differences in cell lineages rather than biological aggressiveness. These included overexpression of FCRL1, MS4A1, PAX5, CD19, FCRLA, TNFRSF17, MEF2B and EAF2 in the B cell lymphoma group and overexpression of AIFM3 and TARP in the LGL lymphoma group.

### 4.6 Differentially expressed genes across lymphoma group pairs

When comparing the top 10 differentially expressed genes across lymphoma group pairs in this study (e.g., small T cell vs. T LGL; CD56+ B cell vs. NK LGL; CD56− B cell vs. T LGL etc) most differentially expressed genes have been already addressed in the context of their biological aggressiveness. The most significant finding from the differential gene expression analysis was the clear molecular distinction between small T cell lymphoma and T cell LGL lymphoma, supporting their divergent cellular origins within the gastrointestinal tract. This comparison provides a strong histogenic evidence that these neoplasms arise from different T lymphocytes residing in the gastrointestinal tract. When comparing DEGs between these groups, CD4 was overexpressed by the small T cell lymphoma group, suggesting a CD4+ T helper cell origin for this neoplasm as described above. In contrast, the T cell LGL group overexpressed the γTCR (TRG) gene with no expression of CD4 or CD8, suggesting this neoplasm arising from γδT cells likely residing in the mucosal lining as cytotoxic intraepithelial lymphocytes. This aligns with previous studies describing separate origins for feline small and large T cell intestinal lymphomas (97), but here provide transcriptional evidence supporting that distinction. Importantly, γδT cells are involved in both innate and adaptive immune response, often with NK-like cytotoxic effects particularly targeting transformed neoplastic cells (98). This could partially explain the increased biologic aggressiveness of T cell LGLs compared to the small T cell lymphoma counterparts due to their innate cytotoxicity actions, thereby linking cell of origin with clinical behaviour.

Of interest, several genes traditionally associated with neural tissues were found to be overexpressed in the NK LGL lymphoma group. These genes included glial cells missing transcription factor 2 (GCM2) and neuromedin U receptor 1 (NMUR1). NK cells are also well known to express CD56, otherwise known as neural cell adhesion molecule 1 that plays an important role in cell adhesion of embryonic neural tissues (3, 6, 99). Although the mechanistic basis for neural-associated gene expression in NK LGLs remains unclear and beyond the scope of this study, NK cells are known to circulate through and interact with the central nervous system, which may partially explain this transcriptional signature (100).

Limitations in this study included the low number of cases analysed as well as incomplete clinical metadata (anatomic location of tumours, signalment and disease progression) that could have further enhanced the analysis and comparison of lymphoma groups. Larger, well-annotated cohorts—ideally integrating molecular profiling with detailed clinical outcomes—are required to validate the genes and pathways identified here and to determine their true prognostic and therapeutic relevance in feline alimentary lymphoma.

An important consideration in the interpretation of these data is that RNA sequencing was performed on whole tissue sections rather than isolated neoplastic cells. As such, the transcriptomic profiles generated represent different signals derived from both malignant lymphocytes and non-neoplastic cellular components of the tumour microenvironment, including reactive T cells, B cells, macrophages, stromal elements and intestinal epithelium. Although the clear clustering of cases according to histologically and immunophenotypically defined subtypes suggests that the dominant transcriptomic signals reflect the neoplastic population, certain pathways—particularly those associated with T-cell activation, immune stimulation and inflammatory signalling—may partially reflect reactive infiltrates rather than neoplastic cells. Indeed, as discussed above, overexpression of B-cell–associated genes in small T-cell lymphoma was most consistent with reactive B-cell aggregates identified histologically, highlighting the influence of the microenvironment on the RNA signature. Therefore, the differential gene expression results should be interpreted as representing tissue-level transcriptional landscapes rather than purely tumour cell–autonomous programmes. Future investigations employing laser capture microdissection or single-cell RNA sequencing, s would allow more precise delineation of neoplastic versus microenvironment-derived signals and further clarify the biological mechanisms underlying these transcriptomic differences.

## 5 Conclusion

Principal component analysis (PCA) of differential gene expression in feline alimentary lymphoma cases revealed distinct clustering based on the lymphoma type, with only a small number of outlier cases. In most subtypes, these outliers were attributable to artifactual data differences. However, the CD56− B cell lymphoma outlier may reflect true biological variation, potentially representing a slightly different subtype of DLBCL or a tumour arising from a different segment of the intestinal tract compared to the clustered CD56− B cell lymphoma cases. These findings emphasise that the utility of gene expression patterns in the classification of feline lymphomas, offering insights beyond morphology and immunophenotype into underlying mechanistic pathogenesis and potential therapy targets. Gene ontology analysis suggested that differences in biologic aggressiveness of feline alimentary lymphomas are related to ECM regulation and cellular growth/metabolism and differential gene expression analysis further identified several genes potentially contributing to the biological behaviour of each lymphoma subtype. Several genes emerged as strong candidates underpinning these behavioural differences. The high aggressiveness observed of feline alimentary LGL lymphomas may be partially explained by overexpression of matrix altering/pro-angiogenic ADAMTS genes (ADAMTS14 and ADAMTSL2), transcription factors (FOXI3, ELAVL2), cell signalling receptors (CCR1, GCGR, NMUR1) and cellular metabolism related genes (MARC1, SLC29A4). The aggressive phenotype of feline alimentary B cell lymphomas may be due to overexpression of chromosomal separating (CENPF) and transcription factor (IGF2BP3) genes, whilst the indolent biologic behaviour of feline alimentary small T cell lymphomas may be due to underexpression of the above listed genes and overexpression of several tumour suppressor genes (RAB17, SYNPO2, GRM4). Collectively, these genes represent promising future targets for diagnosis, prognostication and/or immunotherapy of feline alimentary lymphoma. Importantly, the present study identifies transcriptomic associations rather than establishing causative oncogenic drivers. Whether these differentially expressed genes represent primary driver events, downstream consequences of subtype-specific biology, or reflections of tumour microenvironment interactions remains to be determined. Functional validation studies, integration with genomic mutation analyses, and correlation with detailed clinical outcome data in larger, well-annotated cohorts will be required to clarify their mechanistic and prognostic significance. Comparative analysis between phenotypic group pairs provided further evidence as to the cell of origin of feline alimentary small T cell lymphomas, T cell LGLs and NK cells. The data suggested that small T cell lymphomas were likely derived from a CD4+ T helper cell, whilst T cell LGLs were deriving from intraepithelial cytotoxic γδ (CD4-/CD8-) T cells. This may partially underpin the marked difference in biologic behaviour between these lymphoma types. Overall, the study highlights distinct and biologically meaningful transcriptomic signatures amongst feline alimentary lymphoma subtypes. These findings advance our understanding of lymphoma classification and pathogenesis in cats and provide a foundation for future translational research aimed at improving diagnostic accuracy and therapeutic precision.

## Data Availability

The data presented in the study are deposited in the Figshare repository, accession number 10.6084/m9.figshare.32005221.

## Footnotes

1
https://usegalaxy.org.au/

2
https://hgdownload.soe.ucsc.edu/goldenPath/felCat9/bigZips/genes/

3
https://ftp.ncbi.nlm.nih.gov/genomes/all/GCF/018/350/175/GCF_018350175.1_F.catus_Fca126_mat1.0/
