# Supplementary material for: Transcriptomic and differential gene analysis investigating the differences in biological behaviour between subtypes of feline alimentary lymphoma
Source: Front Vet Sci. 2026 May 29;13:1764747. doi: 10.3389/fvets.2026.1764747 (PMC13259987; doi:10.3389/fvets.2026.1764747)
Supplement: Supplementary file 1 [file Data_Sheet_1.DOCX]

Supplementary Table 1. List of the top 20 DEGs between the CD56- B cell lymphoma outlier (PP_548_09A) and the other CD56- B cell lymphoma cases. Red highlights overexpressed genes, while blue represents underexpressed genes in the outlier case compared to the rest of the CD56- B cell lymphoma group. Unassigned loci are not highlighted.

| **Gene ID** | **Gene name** | **Base mean** | **log2(FC)** | **stdErr** | **Wald-stats** | **P-value** | **adj P value** |
| --- | --- | --- | --- | --- | --- | --- | --- |
| RGS13 | Regulator of G-protein signalling 13 | 2945.008 | -8.53165 | 0.829845 | -10.281 | 8.58E-25 | 1.29E-20 |
| MUC17 | Mucin 17 | 2337.875 | 8.928484 | 0.911627 | 9.794006 | 1.19E-22 | 9.01E-19 |
| MUC2 | Mucin 2 | 6921.717 | 8.571417 | 0.938256 | 9.135477 | 6.51E-20 | 3.27E-16 |
| LOC109500548 | Unassigned | 6018.334 | -5.6422 | 0.692636 | -8.14598 | 3.76E-16 | 1.42E-12 |
| LOC111559512 | Unassigned | 3760.656 | -6.1612 | 0.764562 | -8.05847 | 7.73E-16 | 2.33E-12 |
| BEND4 | BEN containing domain 4 | 2997.817 | -6.44776 | 0.814977 | -7.91159 | 2.54E-15 | 6.39E-12 |
| LOC105260391 | Unassigned | 809.539 | 6.716128 | 0.872371 | 7.698708 | 1.37E-14 | 2.96E-11 |
| KRT8 | Keratin 8 | 313.929 | 7.742983 | 1.018268 | 7.604072 | 2.87E-14 | 5.41E-11 |
| UNC79 | Unc-79 homolog, NALCN channel complex subunit | 284.3172 | 7.540677 | 1.026509 | 7.345943 | 2.04E-13 | 3.42E-10 |
| HNF4A | Hepatocyte nuclear factor 4 alpha | 268.0362 | 7.417199 | 1.031368 | 7.191611 | 6.40E-13 | 9.66E-10 |
| CLDN2 | Claudin 2 | 266.2879 | 7.403351 | 1.031904 | 7.174455 | 7.26E-13 | 9.95E-10 |
| LOC111558287 | Unassigned | 260.0596 | 7.353024 | 1.033836 | 7.112368 | 1.14E-12 | 1.43E-09 |
| LOC101084956 | Unassigned | 289.9907 | 7.006639 | 1.009669 | 6.939544 | 3.93E-12 | 4.56E-09 |
| LOC111560210 | Unassigned | 661.0511 | -5.60788 | 0.82038 | -6.83571 | 8.16E-12 | 8.79E-09 |
| LOC111561459 | Unassigned | 2293.272 | 6.703463 | 0.99594 | 6.730787 | 1.69E-11 | 1.70E-08 |
| LOC109496543 | Unassigned | 1665.842 | 6.71039 | 1.000849 | 6.704698 | 2.02E-11 | 1.90E-08 |
| BPI | Bactericidal permeability increasing | 219.8487 | 6.984395 | 1.04714 | 6.669971 | 2.56E-11 | 2.27E-08 |
| FCRL1 | Fragment crystallisable receptor like 1 | 1514.165 | -4.81253 | 0.723184 | -6.65464 | 2.84E-11 | 2.38E-08 |
| CLCA4 | Chloride channel accessory 4 | 248.2697 | 6.629856 | 1.005564 | 6.593169 | 4.31E-11 | 3.42E-08 |
| GABRA4 | Gamma-aminobutyric acid type A receptor subunit alpha 4 | 646.5166 | -5.22597 | 0.836117 | -6.25028 | 4.10E-10 | 3.09E-07 |

Supplementary Table 2. List of the top 10 DEGs between the small T cell lymphoma group and the CD56- B cell lymphoma group. Red highlights overexpressed genes, while blue represents underexpressed genes in the small T cell lymphoma group compared to the CD56- B cell lymphoma group. Unassigned loci are not highlighted.

| **Gene ID** | **Gene name** | **Base mean** | **Log2 (FC)** | **stdErr** | **Wald-stats** | **P-value** | **adj P value** |
| --- | --- | --- | --- | --- | --- | --- | --- |
| ITGB7 | Integrin subunit beta 7 | 1158.916 | 3.987114 | 0.458097 | 8.703644 | 3.21E-18 | 6.22E-14 |
| LOC111557870 | Unassigned | 70.32363 | 8.241428 | 0.99933 | 8.246956 | 1.62E-16 | 1.57E-12 |
| LAT | Linker for activation of T cells | 1148.748 | 3.681114 | 0.470698 | 7.820551 | 5.26E-15 | 3.40E-11 |
| ITK | IL2 inducible T cell kinase | 885.5407 | 3.811382 | 0.490214 | 7.774933 | 7.55E-15 | 3.65E-11 |
| LOC102899130 | Unassigned | 542.7877 | 6.009067 | 0.776796 | 7.735713 | 1.03E-14 | 3.98E-11 |
| CENPF | Centromere protein F | 2306.248 | -3.78508 | 0.492882 | -7.67948 | 1.60E-14 | 5.16E-11 |
| ADGRG5 | Adhesion G-protein coupled receptor 5 | 1679.718 | 3.264026 | 0.432933 | 7.539333 | 4.72E-14 | 1.31E-10 |
| CCR9 | C-C motif chemokine receptor 9 | 800.7123 | 7.695047 | 1.037606 | 7.416153 | 1.21E-13 | 2.92E-10 |
| GREB1 | Growth regulating oestrogen receptor binding 1 | 305.7673 | 7.13519 | 0.986754 | 7.230974 | 4.80E-13 | 1.03E-09 |
| LOC101097478 | Unassigned | 333.3435 | 7.477606 | 1.044993 | 7.155653 | 8.33E-13 | 1.61E-09 |

Supplementary Table 3. List of the top 10 DEGs between the small T cell lymphoma group and the CD56+ B cell lymphoma group. Red highlights overexpressed genes in the small T cell lymphoma group compared to the CD56+ B cell lymphoma group. Unassigned loci are not highlighted.

| **Gene ID** | **Gene name** | **Base mean** | **log2(FC)** | **stdErr** | **Wald-stats** | **P-value** | **adj P value** |
| --- | --- | --- | --- | --- | --- | --- | --- |
| LOC101090899 | Unassigned | 1355.906 | 10.30235 | 1.350437 | 7.6289 | 2.37E-14 | 3.64E-10 |
| CPNE5 | Copine 5 | 454.1708 | 9.161663 | 1.319546 | 6.94304 | 3.84E-12 | 2.95E-08 |
| TCF7 | Transcription factor 7 | 942.2186 | 9.070392 | 1.320208 | 6.870425 | 6.40E-12 | 3.28E-08 |
| CCR9 | C-C motif chemokine receptor 9 | 291.6592 | 8.677082 | 1.310651 | 6.620435 | 3.58E-11 | 1.38E-07 |
| UST | Uronyl-2-sulfotransferase | 237.9074 | 8.438856 | 1.310294 | 6.440429 | 1.19E-10 | 3.66E-07 |
| RNF125 | Ring finger protein 125 | 203.6239 | 8.229382 | 1.318186 | 6.242959 | 4.29E-10 | 1.10E-06 |
| CCL22 | C-C motif chemokine ligand 22 | 351.0416 | 8.58215 | 1.394011 | 6.156446 | 7.44E-10 | 1.27E-06 |
| LOC102899130 | Unassigned | 198.2437 | 8.171791 | 1.325656 | 6.164338 | 7.08E-10 | 1.27E-06 |
| LOC109492514 | Unassigned | 182.8704 | 7.504264 | 1.218476 | 6.158728 | 7.33E-10 | 1.27E-06 |
| PRKCQ | Protein kinase C theta | 172.4918 | 7.903715 | 1.292136 | 6.116781 | 9.55E-10 | 1.47E-06 |

Supplementary Table 4. List of the top 10 DEGs between the small T cell lymphoma group and the NK LGL lymphoma group. Red highlights overexpressed genes, while blue represents underexpressed genes in the small T cell lymphoma group compared to the NK LGL lymphoma group. Unassigned loci are not highlighted.

| **Gene ID** | **Gene name** | **Base mean** | **Log2 (FC)** | **stdErr** | **Wald-stats** | **P-value** | **adj P value** |
| --- | --- | --- | --- | --- | --- | --- | --- |
| CSN2 | Casein beta | 1462.233 | -10.9642 | 1.363982 | -8.03841 | 9.10E-16 | 1.67E-11 |
| CD4 | Cluster of Differentiation 4 | 967.516 | 8.290763 | 1.162025 | 7.134753 | 9.70E-13 | 8.89E-09 |
| LOC101097478 | Unassigned | 307.0655 | 9.072331 | 1.28524 | 7.058863 | 1.68E-12 | 1.03E-08 |
| GCM2 | Glial cells missing transcription factor 2 | 146.1362 | -8.89484 | 1.315172 | -6.76325 | 1.35E-11 | 6.18E-08 |
| FCRL1 | Fc receptor like 1 | 310.6303 | 7.859518 | 1.225898 | 6.411233 | 1.44E-10 | 4.55E-07 |
| FOXI3 | Forkhead box I3 | 121.4272 | -8.58145 | 1.339477 | -6.40657 | 1.49E-10 | 4.55E-07 |
| LOC109491466 | Unassigned | 153.9953 | 8.270068 | 1.302909 | 6.347386 | 2.19E-10 | 5.74E-07 |
| RNF39 | Ring finger protein 39 | 247.2263 | 6.508175 | 1.036458 | 6.279248 | 3.40E-10 | 7.80E-07 |
| MS4A1 | Membrane spanning 4 domains-A1 | 176.7278 | 7.63372 | 1.244638 | 6.133285 | 8.61E-10 | 1.50E-06 |
| KCNK10 | Potassium two pore domain channel subfamily K member 10 | 58.02436 | 7.552585 | 1.229143 | 6.144593 | 8.02E-10 | 1.50E-06 |

Supplementary Table 5. List of the top 10 DEGs between the small T cell lymphoma group and the T cell LGL lymphoma group. Red highlights overexpressed genes in the small T cell lymphoma group compared to the T cell LGL lymphoma group. Unassigned loci are not highlighted.

| **Gene ID** | **Gene name** | **Base mean** | **log2(FC)** | **stdErr** | **Wald-stats** | **P-value** | **adj P value** |
| --- | --- | --- | --- | --- | --- | --- | --- |
| JCHAIN | Joining chain of multimeric IgA and IgM | 1520.971 | 10.01302 | 1.122 | 8.924258 | 4.49E-19 | 8.25E-15 |
| LOC109497182 | Unassigned | 514.1964 | 11.01303 | 1.258212 | 8.752923 | 2.08E-18 | 1.91E-14 |
| CD4 | Cluster of Differentiation 4 | 619.7173 | 11.07538 | 1.337164 | 8.282737 | 1.20E-16 | 7.38E-13 |
| PDCD1LG2 | Programmed cell death 1 ligand 2 | 379.5082 | 9.820454 | 1.223381 | 8.027304 | 9.96E-16 | 4.58E-12 |
| LOC105260586 | Unassigned | 279.8509 | 8.948692 | 1.144307 | 7.820187 | 5.27E-15 | 1.94E-11 |
| LOC102901032 | Unassigned | 129.9946 | -7.02074 | 0.928647 | -7.56018 | 4.03E-14 | 1.23E-10 |
| COL14A1 | Collagen type 14 alpha chain 1 | 229.6765 | 8.648945 | 1.1678 | 7.406188 | 1.30E-13 | 2.99E-10 |
| LUM | Lumican | 160.9465 | 8.787038 | 1.184135 | 7.420637 | 1.17E-13 | 2.99E-10 |
| LOC102899673 | Unassigned | 282.3735 | 7.861491 | 1.068193 | 7.359616 | 1.84E-13 | 3.77E-10 |
| RNF150 | Ring finger protein 150 | 135.4096 | 7.491805 | 1.024229 | 7.314577 | 2.58E-13 | 4.75E-10 |

Supplementary Table 6. List of the top 10 DEGs between the CD56- B cell lymphoma group and the CD56+ B cell lymphoma group. Red highlights overexpressed genes in the CD56- B cell lymphoma group compared to the CD56+ B cell lymphoma group. Unassigned loci are not highlighted.

| **Gene ID** | **Gene name** | **Base mean** | **Log2 (FC)** | **stdErr** | **Wald-stats** | **P-value** | **adj P value** |
| --- | --- | --- | --- | --- | --- | --- | --- |
| LOC102901324 | Unassigned | 82.22449 | 6.226117 | 1.20309 | 5.175106 | 2.28E-07 | 0.000740667 |
| RNF125 | Ring finger protein 125 | 88.0251 | 6.740785 | 1.247468 | 5.403572 | 6.53E-08 | 0.000740667 |
| FAM210A | Family with sequence similarity 210 family A | 66.85769 | 6.410349 | 1.253928 | 5.112213 | 3.18E-07 | 0.000740667 |
| RPH3A | Rabphilin 3A | 90.3536 | 6.687097 | 1.268818 | 5.270337 | 1.36E-07 | 0.000740667 |
| LOC109492980 | Unassigned | 72.26785 | 6.503583 | 1.252102 | 5.194133 | 2.06E-07 | 0.000740667 |
| LOC109502602 | Unassigned | 77.62669 | 6.517381 | 1.269355 | 5.134404 | 2.83E-07 | 0.000740667 |
| LOC102899458 | Unassigned | 71.35578 | 6.29813 | 1.302348 | 4.83598 | 1.32E-06 | 0.001744543 |
| CDKN3 | Cyclin dependent kinase inhibitor 3 | 48.86868 | 6.062921 | 1.253526 | 4.836693 | 1.32E-06 | 0.001744543 |
| UST | Uronyl-2-sulfotransferase | 71.60021 | 6.34247 | 1.29211 | 4.908613 | 9.17E-07 | 0.001744543 |
| LOC111556217 | Unassigned | 57.87776 | 6.167313 | 1.276522 | 4.831343 | 1.36E-06 | 0.001744543 |

Supplementary Table 7. List of the top 10 DEGs between the CD56-B cell lymphoma group and the NK LGL lymphoma group. Red highlights overexpressed genes, while blue represents underexpressed genes in the CD56- B cell lymphoma group compared to the NK LGL lymphoma group. Unassigned loci are not highlighted.

| **Gene ID** | **Gene name** | **Base mean** | **log2(FC)** | **stdErr** | **Wald-stats** | **P-value** | **adj P value** |
| --- | --- | --- | --- | --- | --- | --- | --- |
| MS4A1 | Membrane spanning 4 domains-A1 | 2476.12 | 11.13036 | 1.036459 | 10.73883 | 6.69E-27 | 1.14E-22 |
| NMUR1 | Neuromedin U receptor 1 | 728.7327 | -10.1706 | 1.010438 | -10.0655 | 7.85E-24 | 6.69E-20 |
| TNFRSF17 | TNF receptor superfamily member 17 | 559.116 | 9.968582 | 1.157074 | 8.615334 | 6.97E-18 | 3.97E-14 |
| CSN2 | Casein beta | 1675.443 | -11.3519 | 1.327301 | -8.55259 | 1.20E-17 | 5.13E-14 |
| CD19 | Cluster of Differentiation 19 | 789.9185 | 6.412098 | 0.794926 | 8.06628 | 7.25E-16 | 2.47E-12 |
| PAX5 | Paired box 5 | 1860.598 | 8.058888 | 1.003907 | 8.027528 | 9.95E-16 | 2.83E-12 |
| LOC109500548 | Unassigned | 2886.385 | 9.355779 | 1.275769 | 7.33344 | 2.24E-13 | 5.47E-10 |
| FCRL1 | Fc receptor like 1 | 726.8799 | 8.594226 | 1.182306 | 7.269037 | 3.62E-13 | 7.72E-10 |
| ELL3 | Elongation factor for RNA polymerase II 3 | 581.6403 | 9.228701 | 1.284649 | 7.183832 | 6.78E-13 | 1.28E-09 |
| RPH3A | Rabphilin 3A | 234.1759 | 8.724615 | 1.217781 | 7.164352 | 7.82E-13 | 1.33E-09 |

Supplementary Table 8. List of the top 10 DEGs between the CD56-B cell lymphoma group and the T cell LGL lymphoma group. Red highlights overexpressed genes, while blue represents underexpressed genes in the CD56- B cell lymphoma group compared to the T cell LGL lymphoma group. Unassigned loci are not highlighted.

| **Gene ID** | **Gene name** | **Base mean** | **Log2 (FC)** | **stdErr** | **Wald-stats** | **P-value** | **adj P value** |
| --- | --- | --- | --- | --- | --- | --- | --- |
| MS4A1 | Membrane spanning 4 domains-A1 | 1606.142 | 11.34234 | 1.062995 | 10.67017 | 1.40E-26 | 2.39E-22 |
| FCRLA | Fc receptor like A | 3953.06 | 10.15357 | 0.958919 | 10.58856 | 3.37E-26 | 2.87E-22 |
| POU2AF1 | POU class 2 homeobox associated factor 1 | 896.394 | 10.64976 | 1.046489 | 10.17666 | 2.52E-24 | 1.43E-20 |
| IQANK1 | IQ motif and ankyrin repeat containing 1 | 409.2431 | -5.99738 | 0.640373 | -9.36545 | 7.57E-21 | 2.58E-17 |
| JCHAIN | Joining chain of multimeric IgA and IgM | 1361.955 | 9.819508 | 1.048275 | 9.367302 | 7.44E-21 | 2.58E-17 |
| LOC109499325 | Unassigned | 519.4325 | -4.54743 | 0.492699 | -9.22963 | 2.72E-20 | 7.70E-17 |
| ST3GAL1 | ST3 beta galactoside alpha-2,3-sialyltransferase 1 | 4448.386 | -4.43776 | 0.488028 | -9.09326 | 9.61E-20 | 2.34E-16 |
| TSPAN8 | Tetraspanin 8 | 1312.128 | 11.26446 | 1.282445 | 8.783578 | 1.58E-18 | 3.37E-15 |
| SMPD5 | Sphingomyelin phosphodiesterase 5 | 1323.937 | -5.54564 | 0.651475 | -8.51244 | 1.70E-17 | 3.22E-14 |
| LOC109500548 | Unassigned | 1867.538 | 11.0674 | 1.303985 | 8.487366 | 2.11E-17 | 3.60E-14 |

Supplementary Table 9. List of the top 10 DEGs between the CD56+ B cell lymphoma group and the NK LGL lymphoma group. Red highlights overexpressed genes, while blue represents underexpressed genes in the CD56+ B cell lymphoma group compared to the NK LGL lymphoma group. Unassigned loci are not highlighted.

| **Gene ID** | **Gene name** | **Base mean** | **Log2 (FC)** | **stdErr** | **Wald-stats** | **P-value** | **adj P value** |
| --- | --- | --- | --- | --- | --- | --- | --- |
| FCRL1 | Fc receptor like 1 | 401.1119 | 9.474067 | 1.401565 | 6.759632 | 1.38E-11 | 1.77E-07 |
| PACSIN1 | Protein kinase C and casein kinase substrate in neurons 1 | 733.3476 | 7.873404 | 1.303599 | 6.039744 | 1.54E-09 | 7.83E-06 |
| LOC109500548 | Unassigned | 369.9313 | 8.97308 | 1.492477 | 6.012207 | 1.83E-09 | 7.83E-06 |
| TARP | TCR gamma alternate reading frame protein | 908.1178 | -8.64924 | 1.488023 | -5.81257 | 6.15E-09 | 1.97E-05 |
| PAX5 | Paired box 5 | 553.7929 | 7.633397 | 1.390975 | 5.487802 | 4.07E-08 | 0.000104427 |
| GZMB | Granzyme B | 277.8925 | -8.47894 | 1.576632 | -5.37788 | 7.54E-08 | 0.000161163 |
| LOC101090899 | Unassigned | 747.6496 | -9.04611 | 1.698744 | -5.32518 | 1.01E-07 | 0.000184853 |
| SLC29A4 | Solute carrier family 29 member 4 | 300.8617 | -8.33255 | 1.637463 | -5.0887 | 3.61E-07 | 0.000578208 |
| MS4A1 | Membrane spanning 4 domains-A1 | 247.2825 | 8.30755 | 1.659324 | 5.006588 | 5.54E-07 | 0.000789806 |
| TXK | Tyrosine kinase | 191.2032 | -7.66803 | 1.544995 | -4.96315 | 6.94E-07 | 0.000889894 |

Supplementary Table 10. List of the top 10 DEGs between the CD56+ B cell lymphoma group and the T cell LGL lymphoma group. Red highlights overexpressed genes, while blue represents underexpressed genes in the CD56+ B cell lymphoma group compared to the T cell LGL lymphoma group. Unassigned loci are not highlighted.

| **Gene ID** | **Gene name** | **Base mean** | **log2(FC)** | **stdErr** | **Wald-stats** | **P-value** | **adj P value** |
| --- | --- | --- | --- | --- | --- | --- | --- |
| POU2AF1 | POU class 2 homeobox associated factor 1 | 831.5999 | 11.29617 | 1.321131 | 8.550378 | 1.23E-17 | 1.55E-13 |
| LOC109500548 | Unassigned | 277.985 | 9.823738 | 1.306583 | 7.518646 | 5.53E-14 | 3.50E-10 |
| FCMR | Fc mu receptor | 434.4377 | 9.451145 | 1.278239 | 7.393881 | 1.43E-13 | 6.01E-10 |
| FCRL1 | Fc receptor like 1 | 298.5316 | 10.28389 | 1.409303 | 7.297147 | 2.94E-13 | 9.29E-10 |
| LOC102900134 | Unassigned | 744.4093 | 9.648803 | 1.329763 | 7.256034 | 3.99E-13 | 1.01E-09 |
| FCRL3 | Fc receptor like 3 | 1116.959 | 9.565494 | 1.363145 | 7.017225 | 2.26E-12 | 4.67E-09 |
| LIX1 | Limb and CNS expressed 1 | 210.8769 | 9.75827 | 1.394294 | 6.998716 | 2.58E-12 | 4.67E-09 |
| LOC109494820 | Unassigned | 121.8237 | 9.16208 | 1.355774 | 6.757823 | 1.40E-11 | 2.21E-08 |
| ADAMTSL2 | ADAMTS like 2 | 1794.96 | -10.1012 | 1.503021 | -6.72057 | 1.81E-11 | 2.29E-08 |
| PRKCH | Protein kinase C eta type | 1657.974 | -7.38444 | 1.098533 | -6.72209 | 1.79E-11 | 2.29E-08 |

Supplementary Table 11. List of the top 10 DEGs between the NK LGL lymphoma group and the T cell LGL lymphoma group. Red highlights overexpressed genes, while blue represents underexpressed genes in the NK LGL lymphoma group compared to the T cell LGL lymphoma group. Unassigned loci are not highlighted.

| **Gene ID** | **Gene name** | **Base mean** | **log2(FC)** | **stdErr** | **Wald-stats** | **P-value** | **adj P value** |
| --- | --- | --- | --- | --- | --- | --- | --- |
| CSN2 | Casein beta | 996.094 | 9.753217 | 1.425447 | 6.842216 | 7.80E-12 | 6.11E-08 |
| XKR6 | XK related 6 | 212.0996 | -7.69494 | 1.125278 | -6.83825 | 8.02E-12 | 6.11E-08 |
| APP | Amyloid beta precursor protein | 838.6323 | 5.247605 | 0.817642 | 6.417976 | 1.38E-10 | 7.02E-07 |
| PDCD1LG2 | Programmed cell death 1 ligand 2 | 141.3934 | 8.520328 | 1.402766 | 6.073947 | 1.25E-09 | 4.76E-06 |
| LOC101089456 | Unassigned | 88.58815 | 8.660504 | 1.466144 | 5.906994 | 3.48E-09 | 1.06E-05 |
| LOC109496543 | Unassigned | 3025.908 | 8.302822 | 1.438936 | 5.770112 | 7.92E-09 | 2.01E-05 |
| IGF1 | Insulin like growth factor 1 | 406.5445 | 7.181105 | 1.259925 | 5.699631 | 1.20E-08 | 2.29E-05 |
| GCM2 | Glial cells missing transcription factor 2 | 102.3536 | 8.167888 | 1.428279 | 5.718691 | 1.07E-08 | 2.29E-05 |
| LOC109499065 | Unassigned | 178.884 | 6.182129 | 1.089007 | 5.67685 | 1.37E-08 | 2.32E-05 |
| RAB30 | Member of RAS oncogene family 30 | 68.17048 | 8.326917 | 1.473928 | 5.649473 | 1.61E-08 | 2.45E-05 |

Supplementary Table 12. List of the top 20 DEGs between the small T cell lymphoma group and combined LGL lymphoma group. Red highlights overexpressed genes, while blue represents underexpressed genes in the small T cell lymphoma group compared to the combined LGL lymphoma group. Unassigned loci are not highlighted.

| **Gene ID** | **Gene name** | **log2(FC)** | **Wald-stats** | **P-value** | **adj P value** |
| --- | --- | --- | --- | --- | --- |
| FCRL1 | Fc receptor like 1 | 8.446826 | 7.670057 | 1.72E-14 | 3.27E-10 |
| MS4A1 | Membrane spanning 4 domains-A1 | 7.777324 | 7.379611 | 1.59E-13 | 1.51E-09 |
| CD4 | Cluster of Differentiation 4 | 8.909964 | 7.275385 | 3.45E-13 | 2.19E-09 |
| CC1H1orf167 | Chromosome C1 C1orf167 homolog | -7.10947 | -6.65751 | 2.79E-11 | 1.32E-07 |
| ADAMTS14 | ADAM metallopeptidase with thrombospondin type 1 motif 14 | -5.74289 | -6.52261 | 6.91E-11 | 2.63E-07 |
| KCNK10 | Potassium two pore domain channel subfamily K member 10 | 6.795194 | 6.344891 | 2.23E-10 | 7.05E-07 |
| CD40LG | Cluster of Differentiation 40 ligand | 7.654703 | 6.267497 | 3.67E-10 | 9.96E-07 |
| LOC101097478 | Unassigned | 7.294547 | 6.036047 | 1.58E-09 | 3.75E-06 |
| LOC109495826 | Unassigned | -5.12788 | -5.44833 | 5.08E-08 | 9.66E-05 |
| LOC102901843 | Unassigned | 6.698193 | 5.454751 | 4.90E-08 | 9.66E-05 |
| FOXI3 | Forkhead box I3 | -7.03984 | -5.38407 | 7.28E-08 | 0.000126 |
| LOC101098516 | Unassigned | 6.194702 | 5.338657 | 9.36E-08 | 0.000136 |
| MYH11 | Myosin heavy chain 11 | 5.811081 | 5.326433 | 1.00E-07 | 0.000136 |
| CCR1 | C-C motif chemokine receptor 1 | -3.31488 | -5.33669 | 9.47E-08 | 0.000136 |
| PLB1 | Phospholipase B1 | 6.52586 | 5.300977 | 1.15E-07 | 0.000146 |
| LOC111558527 | Unassigned | -6.36802 | -5.21622 | 1.83E-07 | 0.000174 |
| TNFRSF17 | TNF receptor superfamily member 17 | 7.194554 | 5.217365 | 1.81E-07 | 0.000174 |
| GCGR | Glucagon receptor | -6.05665 | -5.22673 | 1.73E-07 | 0.000174 |
| RAB17 | Member of RAS oncogene family member 17 | 6.342436 | 5.22727 | 1.72E-07 | 0.000174 |
| SYNPO2 | Synaptopodin 2 | 5.685646 | 5.247548 | 1.54E-07 | 0.000174 |

Supplementary Table 13. List of the top 20 DEGs between the small T cell lymphoma group and combined B cell lymphoma group. Red highlights overexpressed genes, while blue represents underexpressed genes in the small T cell lymphoma group compared to the combined B cell lymphoma group. Unassigned loci are not highlighted.

| **Gene ID** | **Gene name** | **log2(FC)** | **Wald-stats** | **P-value** | **adj P value** |
| --- | --- | --- | --- | --- | --- |
| IGF2BP3 | Insulin like growth factor 2 mRNA binding protein 3 | -6.01485 | -7.07303 | 1.52E-12 | 2.61E-08 |
| LAT | Linker for activation of T cells | 3.675606 | 6.730352 | 1.69E-11 | 1.46E-07 |
| CENPF | Centromere protein F | -3.64603 | -6.6194 | 3.61E-11 | 1.55E-07 |
| CCR9 | C-C motif chemokine receptor 9 | 7.669428 | 6.644244 | 3.05E-11 | 1.55E-07 |
| AIFM3 | Apoptosis inducing factor mitochondria associated 3 | 5.631636 | 6.56443 | 5.22E-11 | 1.80E-07 |
| MEF2B | Myocyte enhancer factor 2B | -5.95878 | -6.49361 | 8.38E-11 | 2.41E-07 |
| LOC111557870 | Unassigned | 6.810853 | 6.453847 | 1.09E-10 | 2.68E-07 |
| LOC101097478 | Unassigned | 7.284737 | 6.402448 | 1.53E-10 | 3.29E-07 |
| LOC102899854 | Unassigned | 5.147728 | 6.380246 | 1.77E-10 | 3.38E-07 |
| ANKRD34C | Ankyrin repeat domain 34C | -4.94277 | -6.31704 | 2.67E-10 | 4.59E-07 |
| GRM4 | Glutamate metabotropic receptor 4 | 6.40362 | 6.111252 | 9.89E-10 | 1.55E-06 |
| ADGRG5 | Adhesion G protein-coupled receptor G5 | 3.547449 | 6.043783 | 1.51E-09 | 2.16E-06 |
| LOC102899130 | Unassigned | 6.212481 | 5.944424 | 2.77E-09 | 3.68E-06 |
| LOC109497583 | Unassigned | 6.837325 | 5.865528 | 4.48E-09 | 5.51E-06 |
| PNMA6A | Paraneoplastic antigen-like protein 6B | 6.41003 | 5.854054 | 4.80E-09 | 5.51E-06 |
| GDF6 | Growth differentiation factor 6 | 6.496693 | 5.736397 | 9.67E-09 | 9.26E-06 |
| LOC101090899 | Unassigned | 6.794944 | 5.747932 | 9.03E-09 | 9.26E-06 |
| LOC101085707 | Unassigned | 6.946656 | 5.742188 | 9.35E-09 | 9.26E-06 |
| TARP | TCR gamma alternate reading frame protein | 6.474912 | 5.59163 | 2.25E-08 | 1.94E-05 |
| LOC109500548 | Unassigned | -5.83076 | -5.59339 | 2.23E-08 | 1.94E-05 |

Supplementary Table 14. List of the top 20 DEGs between the combined B cell lymphoma group and the combined LGL lymphoma group. Red highlights overexpressed genes, while blue represents underexpressed genes in the combined B cell lymphoma group compared to the combined LGL lymphoma group. Unassigned loci are not highlighted.

| **Gene ID** | **Gene name** | **log2(FC)** | **Wald-stats** | **P-value** | **adj P value** |
| --- | --- | --- | --- | --- | --- |
| LOC109500548 | Unassigned | 10.65135 | 11.15929 | 6.45E-29 | 1.11E-24 |
| FCRL1 | Fc receptor like 1 | 10.02308 | 10.80624 | 3.22E-27 | 2.76E-23 |
| MS4A1 | Membrane spanning 4 domains-A1 | 10.43511 | 10.62587 | 2.26E-26 | 1.29E-22 |
| PAX5 | Paired box 5 | 8.009847 | 9.894057 | 4.42E-23 | 1.90E-19 |
| CD19 | Cluster of Differentiation 19 | 6.289816 | 8.829777 | 1.05E-18 | 3.61E-15 |
| MEF2B | Myocyte enhancer factor 2B | 7.77075 | 8.588065 | 8.84E-18 | 2.53E-14 |
| AIFM3 | Apoptosis inducing factor mitochondria associated 3 | -6.22509 | -8.5546 | 1.18E-17 | 2.91E-14 |
| LOC102900134 | Unassigned | 8.36112 | 8.488264 | 2.10E-17 | 4.01E-14 |
| EAF2 | ELL associated factor 2 | 9.067535 | 8.501924 | 1.86E-17 | 4.01E-14 |
| GCGR | Glucagon receptor | -8.33147 | -8.1893 | 2.63E-16 | 4.52E-13 |
| FCRLA | Fc receptor like A | 8.040197 | 8.174473 | 2.97E-16 | 4.64E-13 |
| TARP | TCR gamma alternate reading frame protein | -7.64042 | -7.9268 | 2.25E-15 | 3.22E-12 |
| MARC1 | Mitochondrial amidoxime reducing component 1 | -7.60822 | -7.77559 | 7.51E-15 | 9.93E-12 |
| TSGA10IP | Testis specific 10 interacting protein | -8.02919 | -7.60649 | 2.82E-14 | 3.46E-11 |
| SLC29A4 | Solute carrier family 29 member 4 | -9.05579 | -7.54702 | 4.45E-14 | 5.10E-11 |
| TNFRSF17 | TNF receptor superfamily member 17 | 8.781265 | 7.199517 | 6.04E-13 | 6.49E-10 |
| GREB1 | Growth regulating oestrogen receptor binding 1 | -8.2373 | -7.17832 | 7.06E-13 | 7.14E-10 |
| NMUR1 | Neuromedin U receptor 1 | -7.84344 | -7.1086 | 1.17E-12 | 1.12E-09 |
| ELAVL2 | ELAV like RNA binding protein 2 | -8.24203 | -7.02093 | 2.20E-12 | 1.99E-09 |
| ADAMTSL2 | ADAMTS like 2 | -7.80453 | -6.9941 | 2.67E-12 | 2.30E-09 |
