# Supplementary material for: Transcriptomic and differential gene analysis investigating the differences in biological behaviour between subtypes of feline alimentary lymphoma
Source: Front Vet Sci. 2026 May 29;13:1764747. doi: 10.3389/fvets.2026.1764747 (PMC13259987; doi:10.3389/fvets.2026.1764747)
Supplement: Supplementary file 2 [file Data_Sheet_2.DOCX]

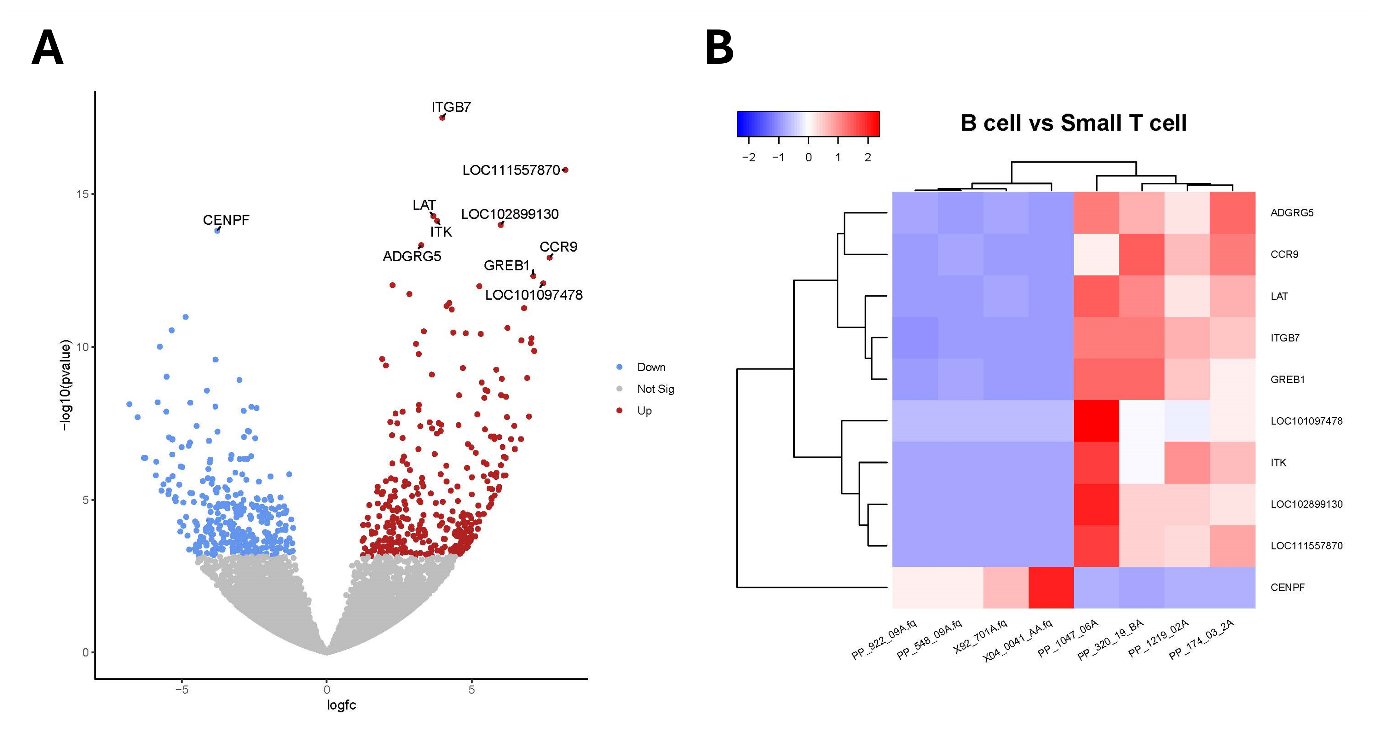


**Supplementary Figure 1.** Volcano plot of differentially expressed genes when comparing the small T cell lymphoma group with the CD56- B cell lymphoma group (A). A heatmap of the top 10 DEGs between these groups (B). Red represents gene overexpression whereas blue represents gene underexpression in the small T cell lymphoma group compared to CD56-B cell lymphoma group.


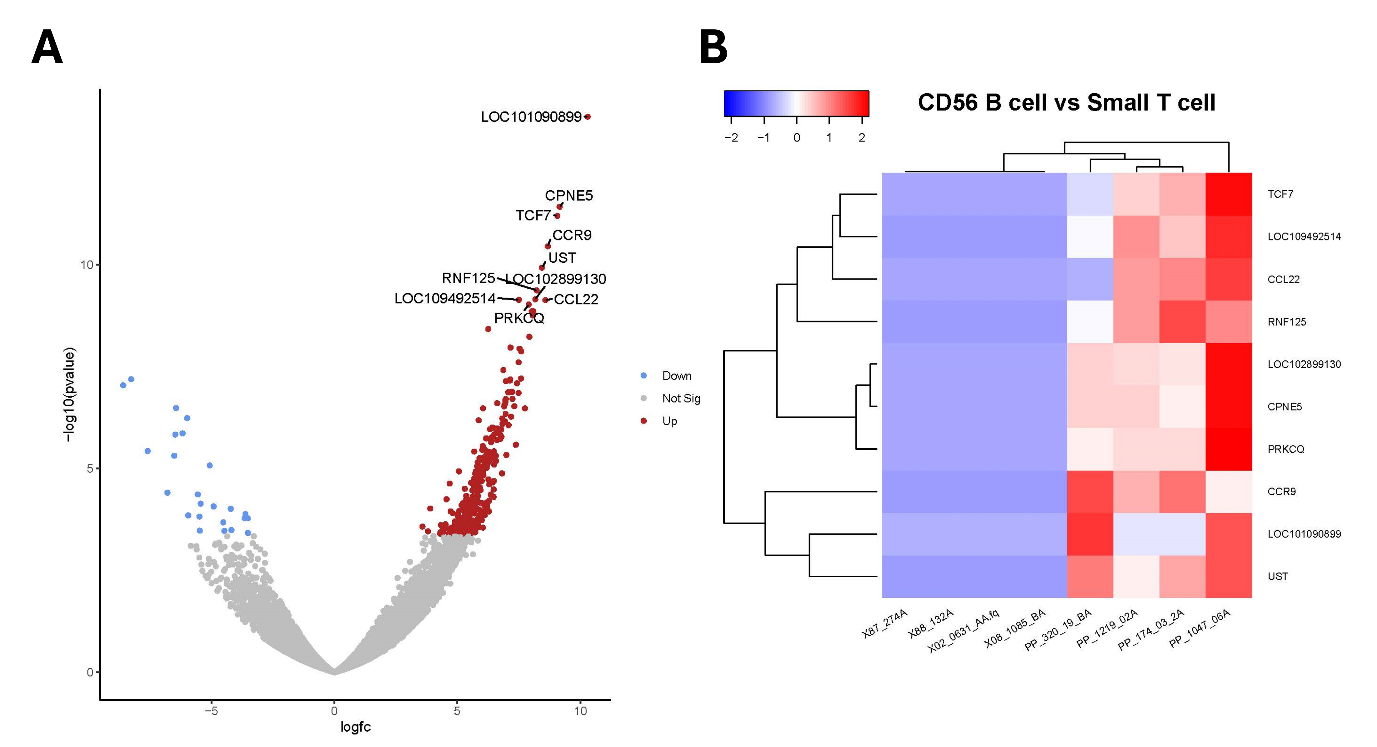


**Supplementary Figure 2.** Volcano plot of differentially expressed genes when comparing the small T cell lymphoma group with the CD56+ B cell lymphoma group (A). A heatmap of the top 10 DEGs between these groups (B). Red represents gene overexpression whereas blue represents gene underexpression in the small T cell lymphoma group compared to CD56+ B cell lymphoma group.


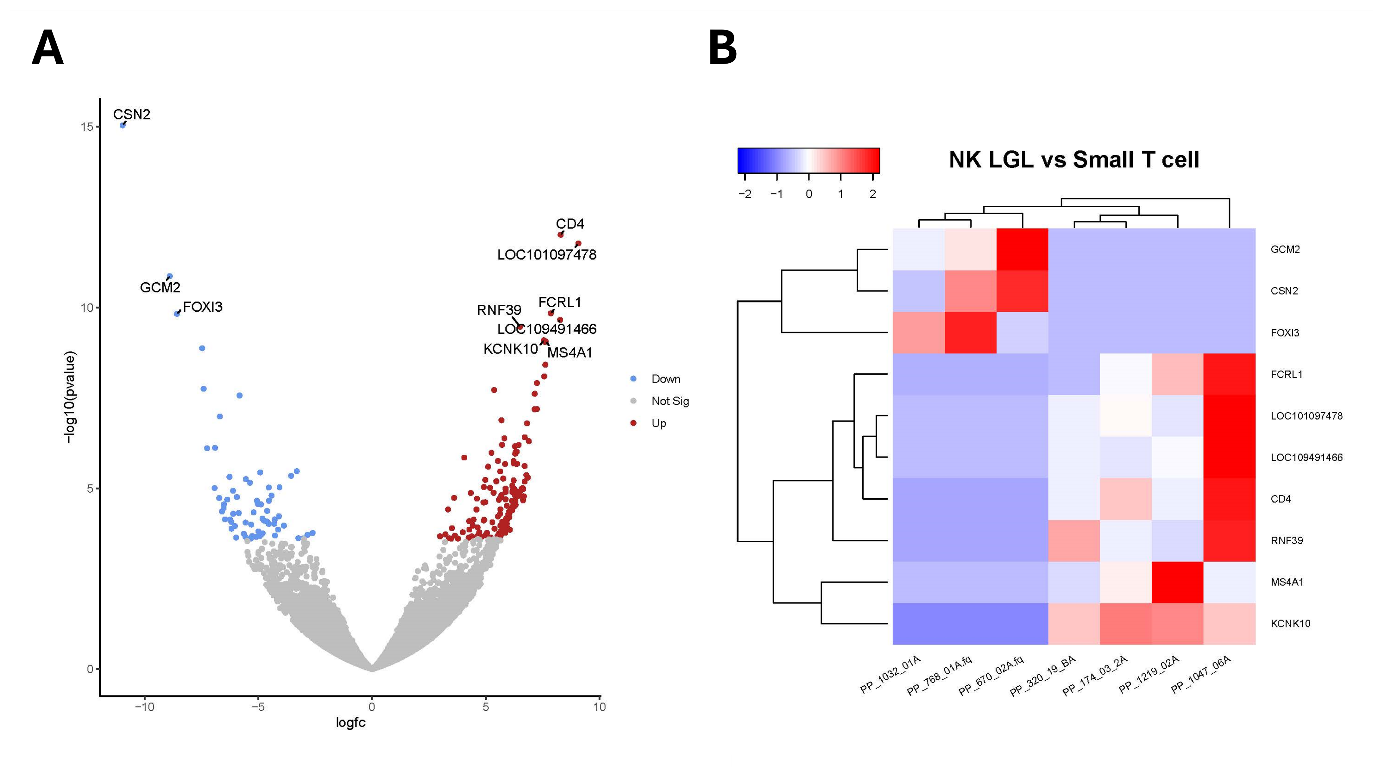


**Supplementary Figure 3.** Volcano plot of differentially expressed genes when comparing the small T cell lymphoma group with the NK LGL lymphoma group (A). A heatmap of the top 10 DEGs between these groups (B). Red represents gene overexpression whereas blue represents gene underexpression in the small T cell lymphoma group compared to NK LGL lymphoma group.


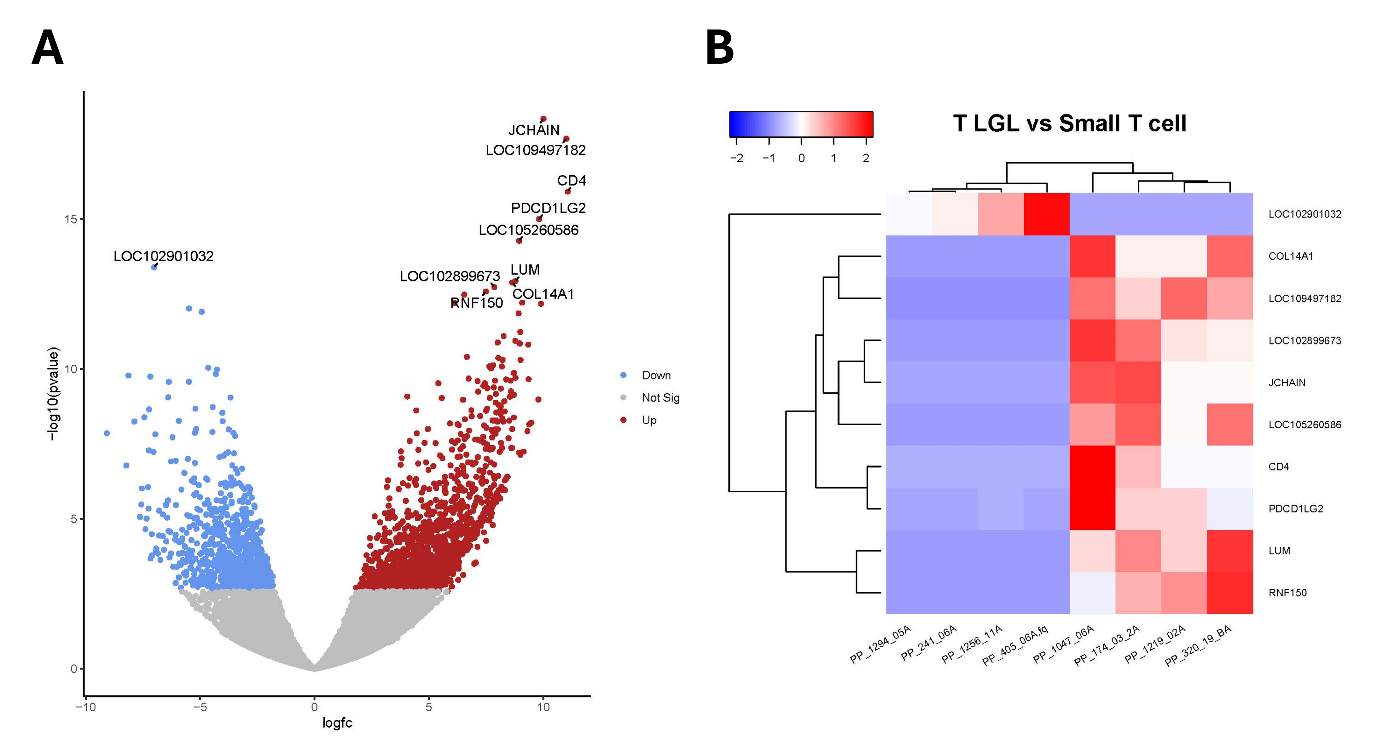


**Supplementary Figure 4.** Volcano plot of differentially expressed genes when comparing the small T cell lymphoma group with the T cell LGL lymphoma group (A). A heatmap of the top 10 DEGs between these groups (B). Red represents gene overexpression whereas blue represents gene underexpression in the small T cell lymphoma group compared to T cell LGL lymphoma group.


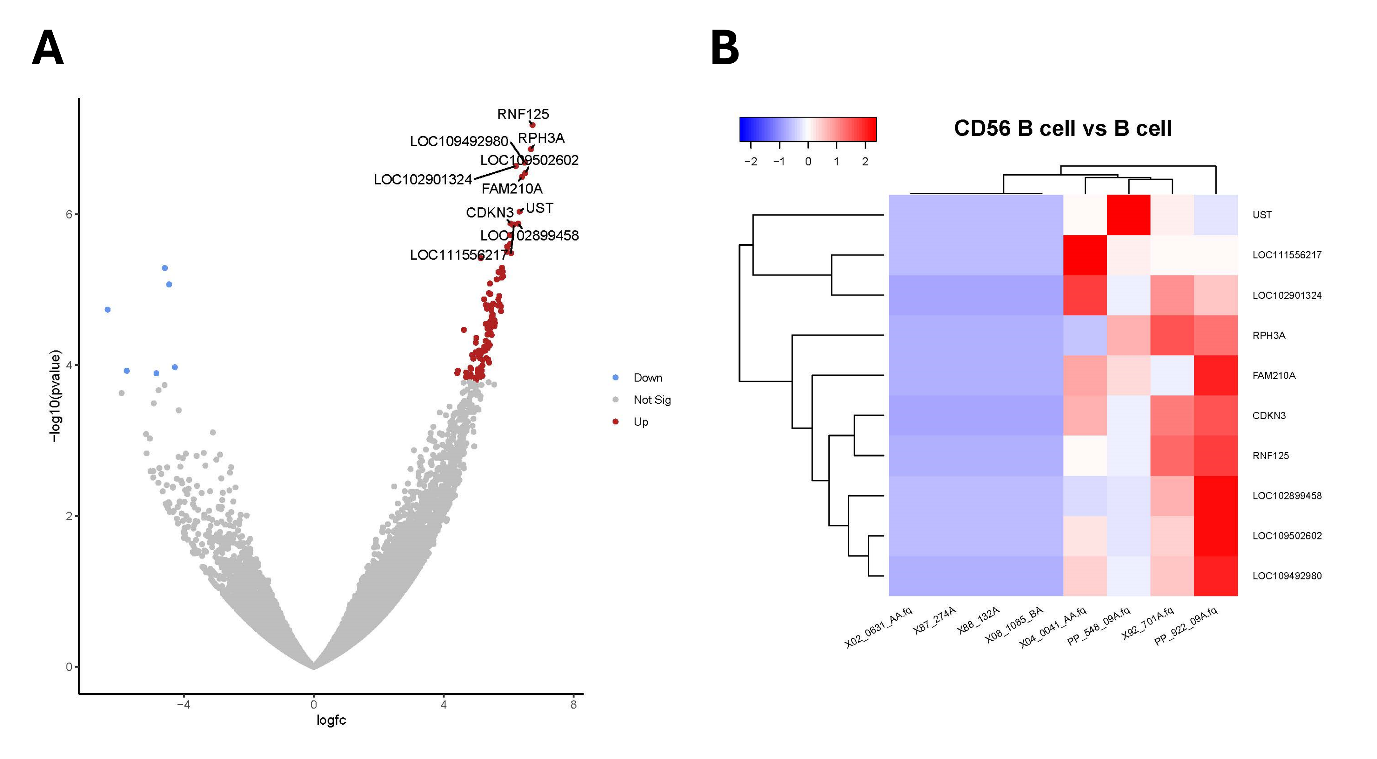


**Supplementary Figure 5.** Volcano plot of differentially expressed genes when comparing the CD56- B cell lymphoma group with the CD56+ B cell lymphoma group (A). A heatmap of the top 10 DEGs between these groups (B). Red represents gene overexpression whereas blue represents gene underexpression in the CD56- B cell lymphoma group compared to CD56+ B cell lymphoma group.


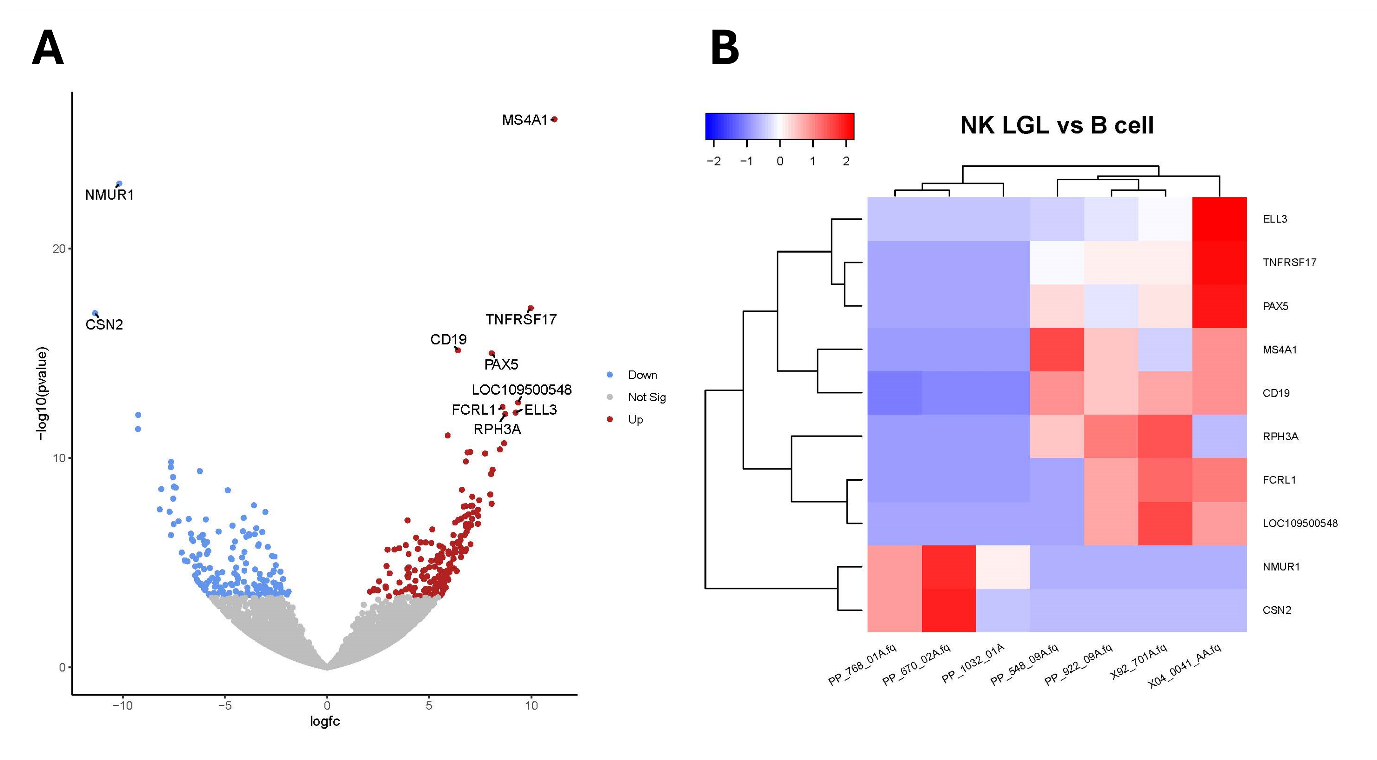


**Supplementary Figure 6.** **V**olcano plot of differentially expressed genes when comparing the CD56-B cell lymphoma group with the NK LGL lymphoma group (A). A heatmap of the top 10 DEGs between these groups (B). Red represents gene overexpression whereas blue represents gene underexpression in the CD56- B cell lymphoma group compared to NK LGL lymphoma group.


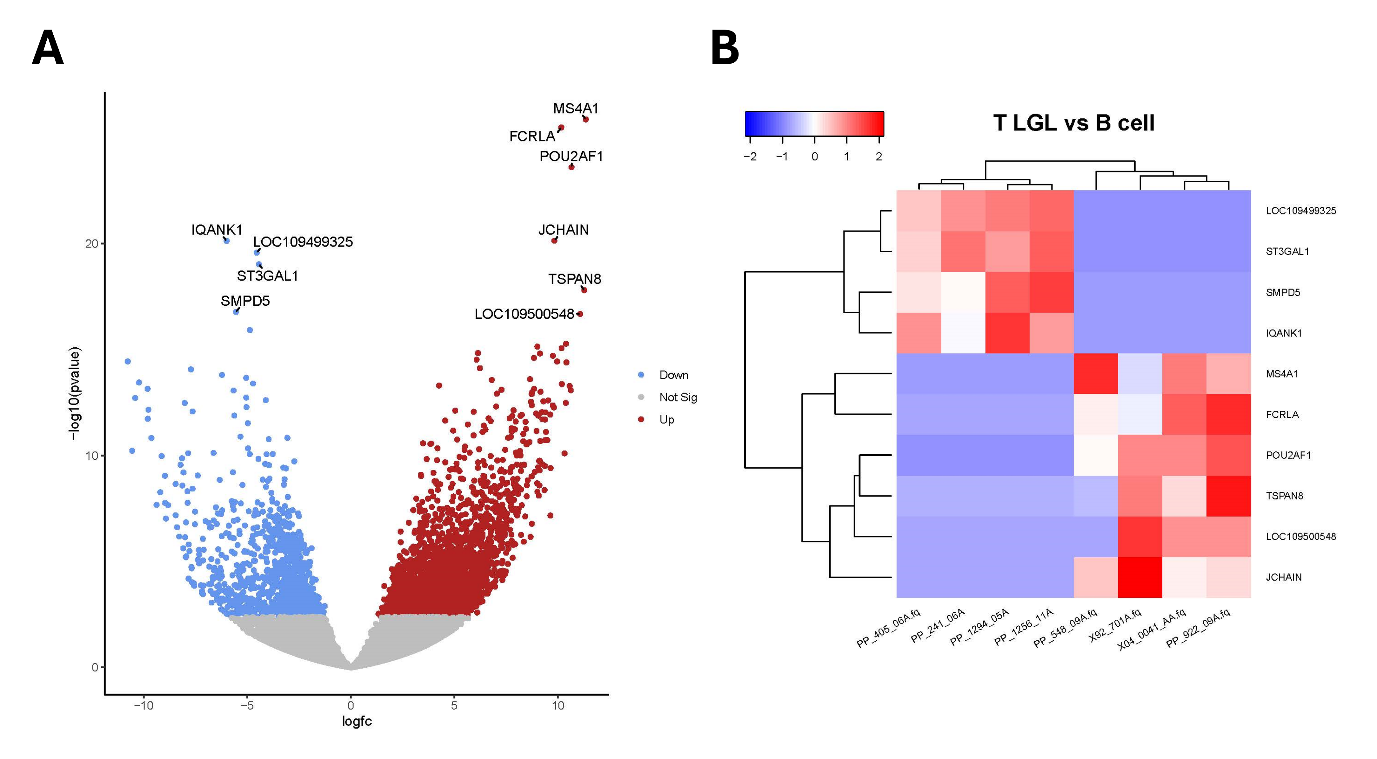


**Supplementary Figure 7.** Volcano plot of differentially expressed genes when comparing the CD56- B cell lymphoma group with the T cell LGL lymphoma group (A). A heatmap of the top 10 DEGs between these groups (B). Red represents gene overexpression whereas blue represents gene underexpression in the CD56- B cell lymphoma group compared to T cell LGL lymphoma group.


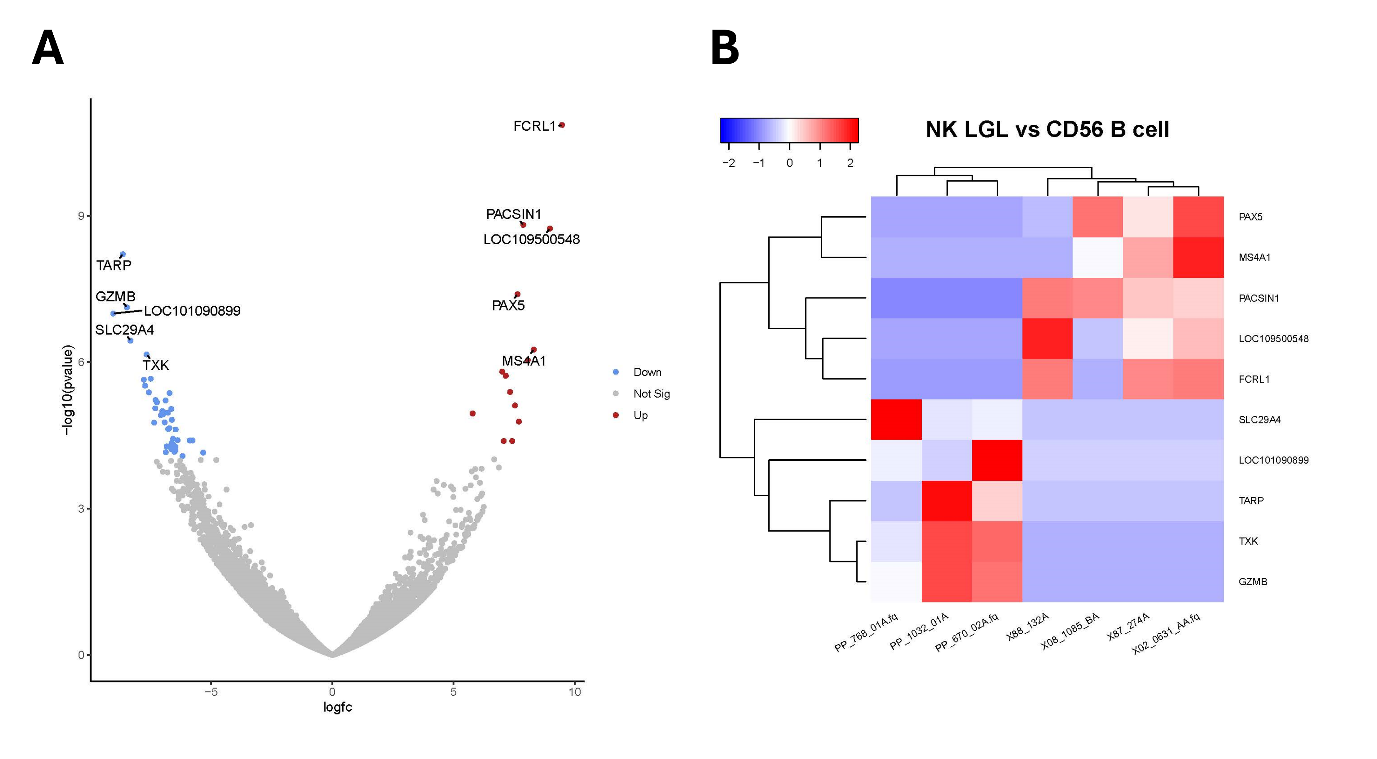


**Supplementary Figure 8.** A volcano plot of differentially expressed genes when comparing the CD56+ B cell lymphoma group with the NK LGL lymphoma group (A). A heatmap of the top 10 DEGs between these groups (B). Red represents gene overexpression whereas blue represents gene underexpression in the CD56+ B cell lymphoma group compared to NK LGL lymphoma group.


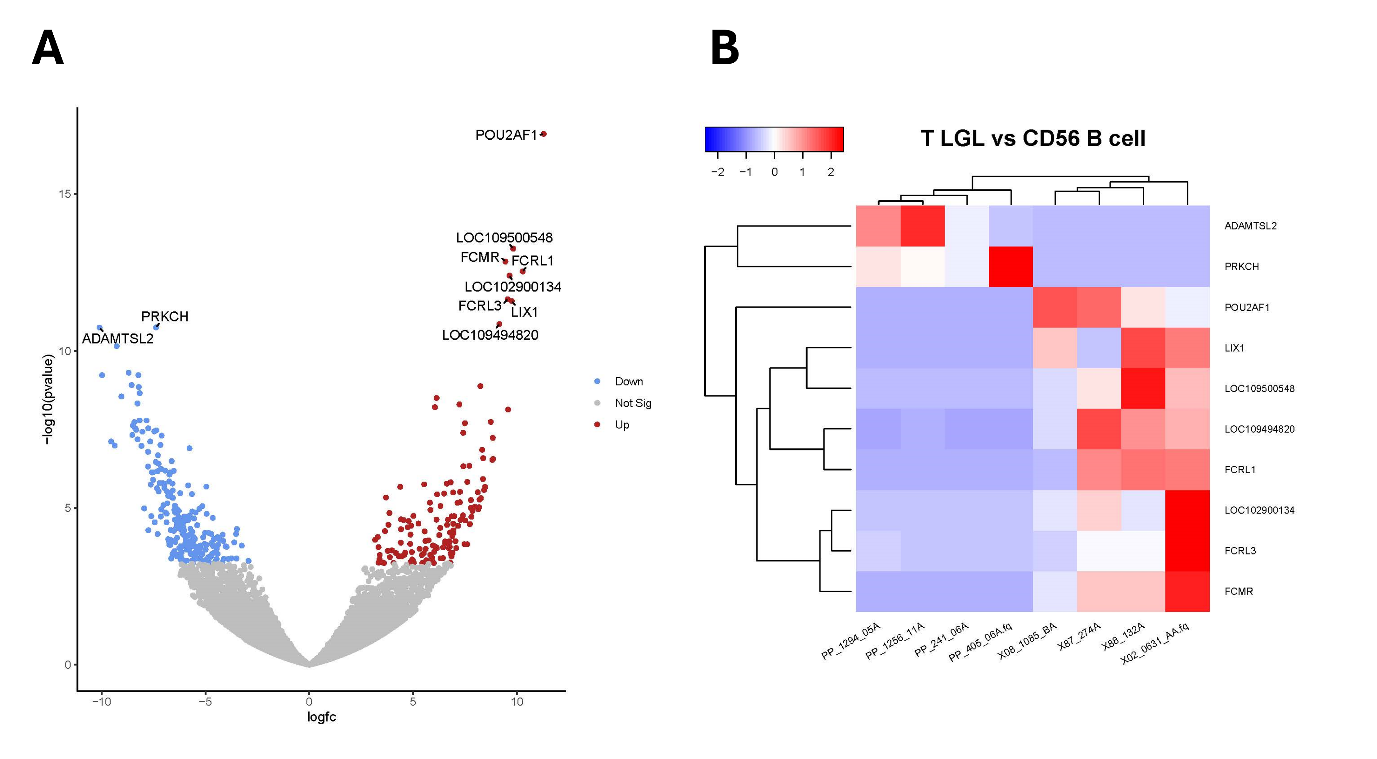


**Supplementary Figure 9.** Volcano plot of differentially expressed genes when comparing the CD56+ B cell lymphoma group with the T cell LGL lymphoma group (A). A heatmap of the top 10 DEGs between these groups (B). Red represents gene overexpression whereas blue represents gene underexpression in the CD56+ B cell lymphoma group compared to T cell LGL lymphoma group.


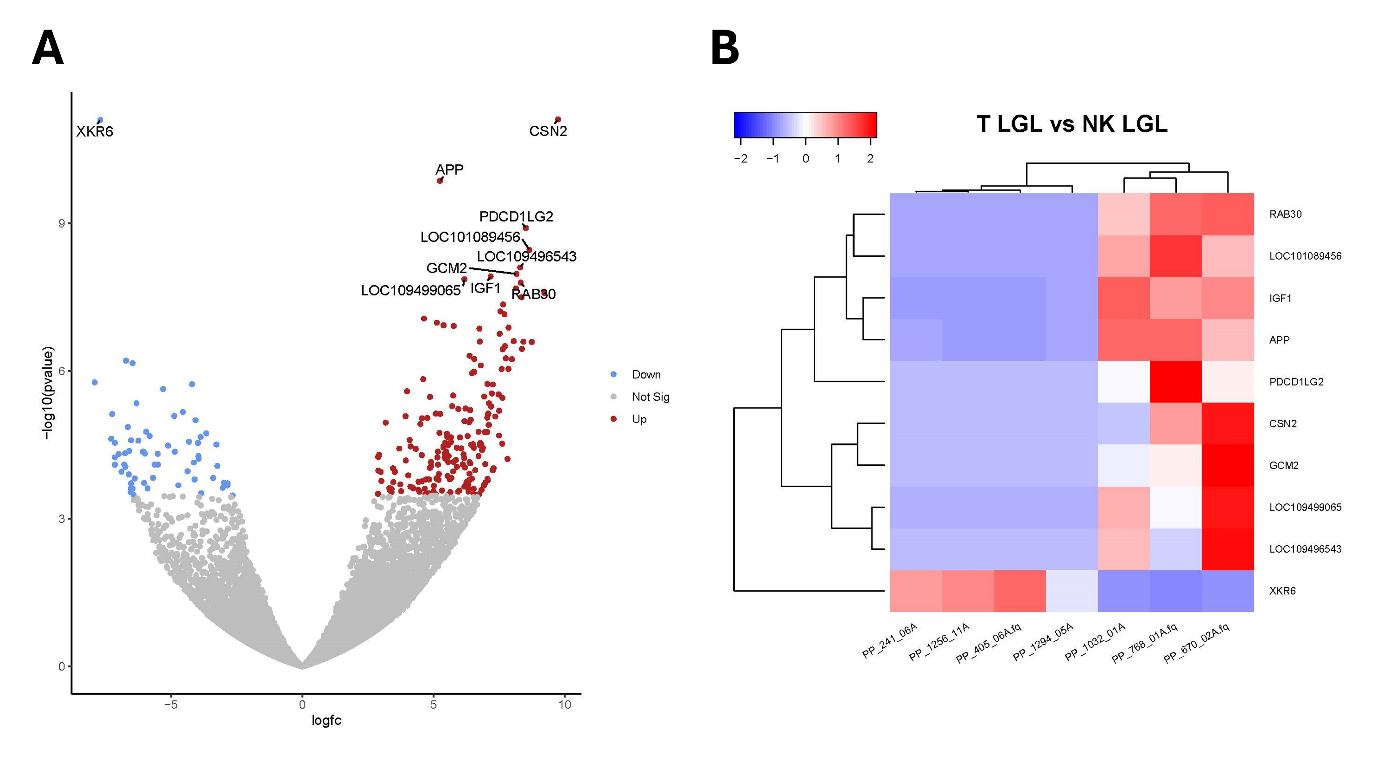


**Supplementary Figure 10.** Volcano plot of differentially expressed genes when comparing the NK LGL lymphoma group with the T cell LGL lymphoma group (A). A heatmap of the top 10 DEGs between these groups (B). Red represents gene overexpression whereas blue represents gene underexpression in the NK LGL lymphoma group compared to T cell LGL lymphoma group.


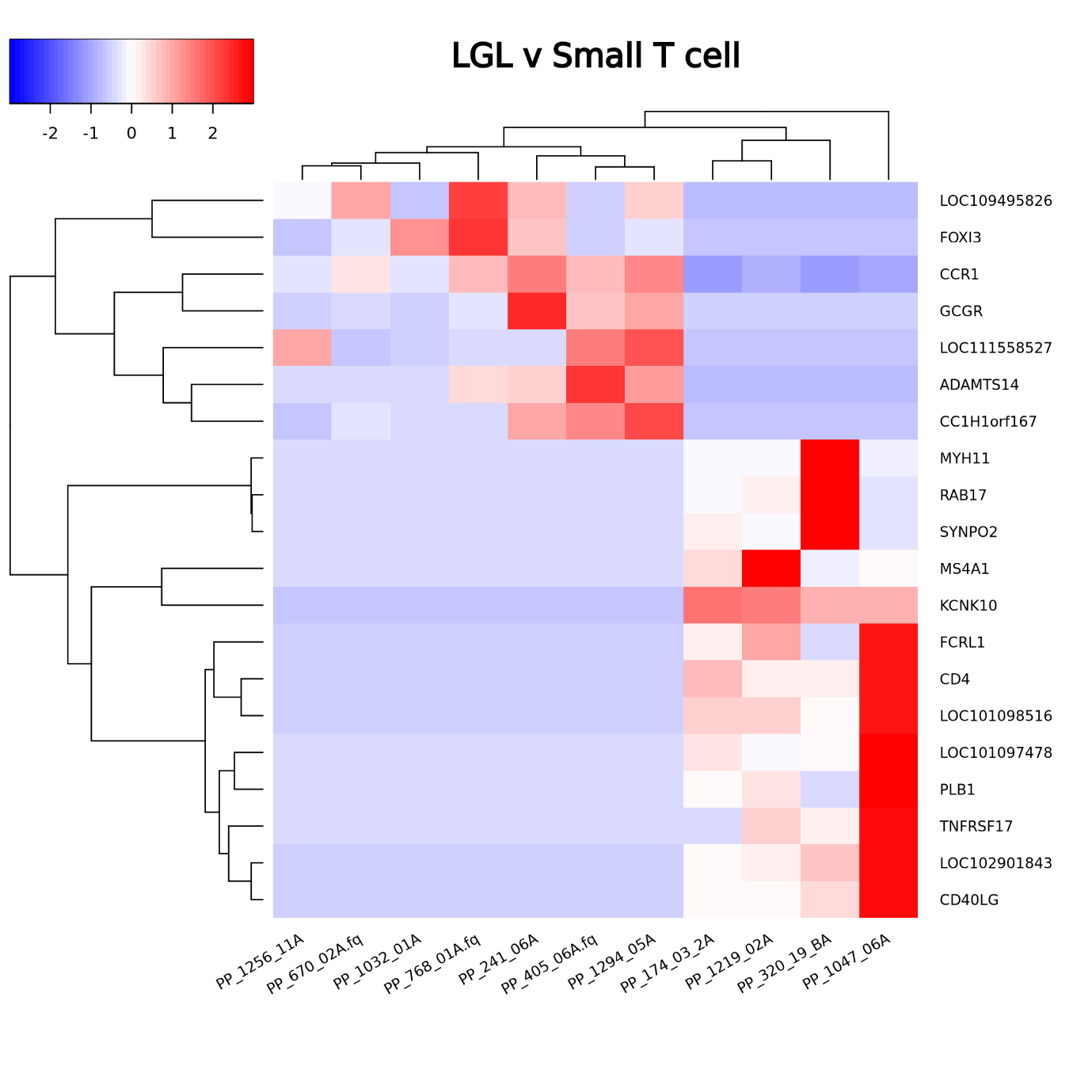


**Supplementary Figure 11.** Heatmap of the top 20 DEGs between small T cell lymphoma group and the combined LGL lymphoma groups. Red represents gene overexpression whereas blue represents gene underexpression in the small T cell lymphoma group compared to combined LGL lymphoma group.


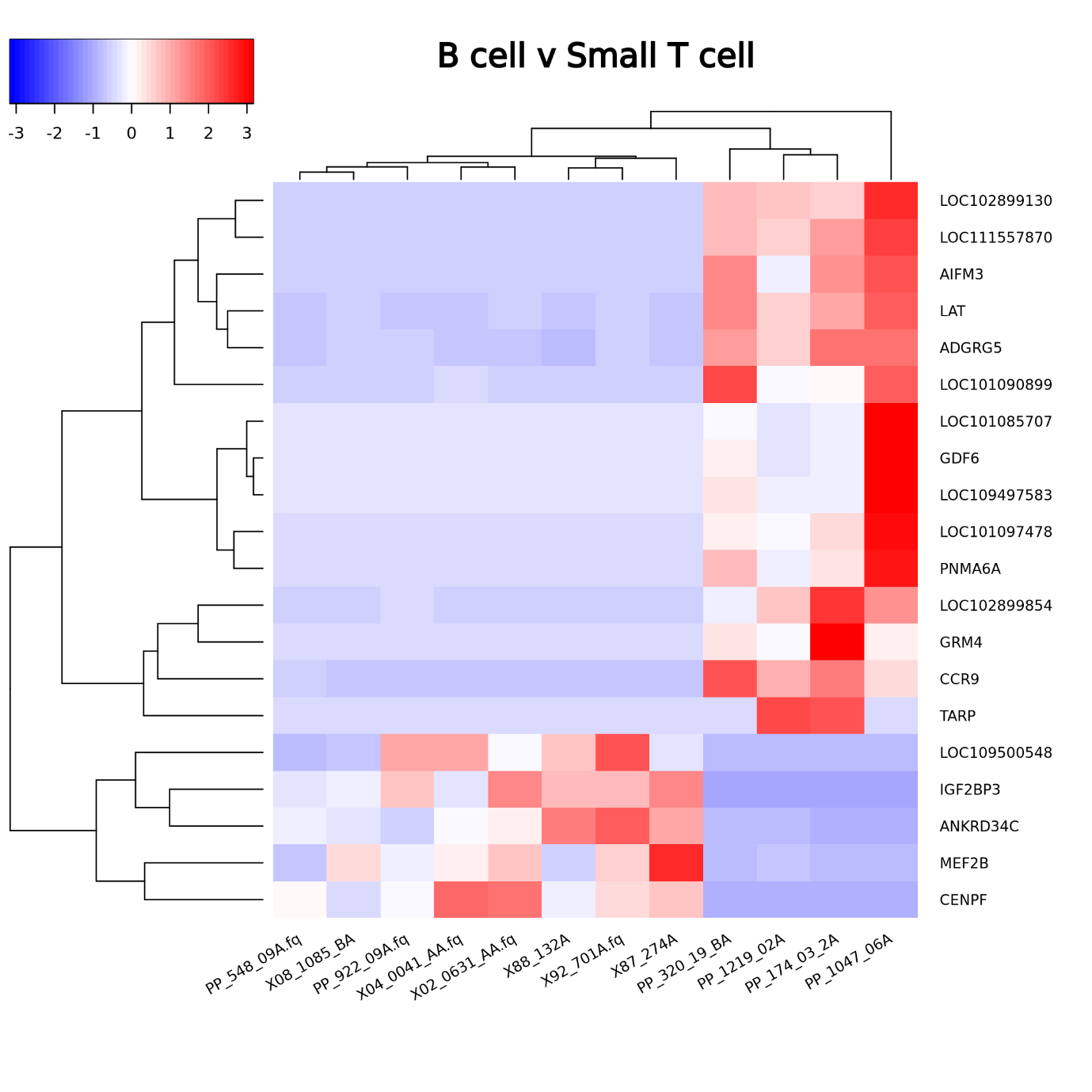


**Supplementary Figure 12.** Heatmap of the top 20 DEGs between small T cell lymphoma group and the combined B cell lymphoma group. Red represents gene overexpression whereas blue represents gene underexpression in the small T cell lymphoma group compared to combined B cell lymphoma group.


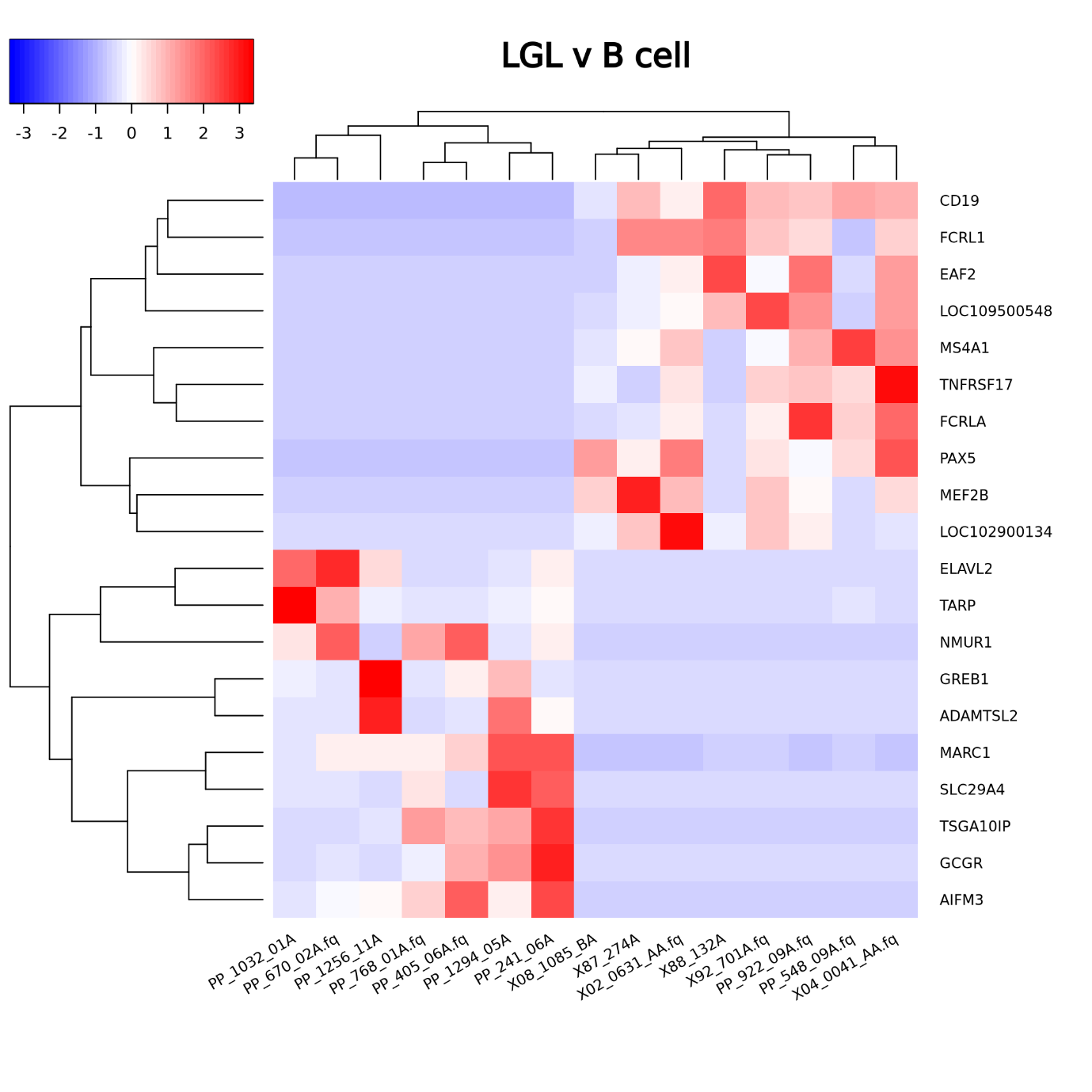


**Supplementary Figure 13.** Heatmap of the top 20 DEGs between the combined B cell lymphoma group and the combined LGL lymphoma group. Red represents gene overexpression whereas blue represents gene underexpression in the combined B cell lymphoma group compared to the combined LGL lymphoma group.
